# Machine Learning Enables Comprehensive Prediction of the Relative Protein Abundance of Multiple Proteins on the Protein Corona

**DOI:** 10.34133/research.0487

**Published:** 2024-09-25

**Authors:** Xiuhao Fu, Chao Yang, Yunyun Su, Chunling Liu, Haoye Qiu, Yanyan Yu, Gaoxing Su, Qingchen Zhang, Leyi Wei, Feifei Cui, Quan Zou, Zilong Zhang

**Affiliations:** ^1^ School of Computer Science and Technology, Hainan University, Haikou 570228, China.; ^2^ School of Pharmacy, Nantong University, Nantong, Jiangsu 226001, China.; ^3^ Centre for Artificial Intelligence Driven Drug Discovery, Faculty of Applied Science, Macao Polytechnic University, Macao SAR, China.; ^4^ School of Informatics, Xiamen University, Xiamen, China.; ^5^ Institute of Fundamental and Frontier Sciences, University of Electronic Science and Technology of China, Chengdu 610054, China.; ^6^ Yangtze Delta Region Institute (Quzhou), University of Electronic Science and Technology of China, Quzhou 324000, China.

## Abstract

Understanding protein corona composition is essential for evaluating their potential applications in biomedicine. Relative protein abundance (RPA), accounting for the total proteins in the corona, is an important parameter for describing the protein corona. For the first time, we comprehensively predicted the RPA of multiple proteins on the protein corona. First, we used multiple machine learning algorithms to predict whether a protein adsorbs to a nanoparticle, which is dichotomous prediction. Then, we selected the top 3 performing machine learning algorithms in dichotomous prediction to predict the specific value of RPA, which is regression prediction. Meanwhile, we analyzed the advantages and disadvantages of different machine learning algorithms for RPA prediction through interpretable analysis. Finally, we mined important features about the RPA prediction, which provided effective suggestions for the preliminary design of protein corona. The service for the prediction of RPA is available at http://www.bioai-lab.com/PC_ML.

## Introduction

The small size of nanoparticles is associated with several unique properties that mediate a profound impact on the development of many technological fields, including medicine [1–3]. The nanoparticles in question are sufficiently small to allow access to almost all areas of the body. This has led to the development of a novel approach to medicine known as nanomedicine [4]. Upon entering biological fluids, nanoparticles interact with a range of biopolymers, including proteins, which bind to them, forming the “protein corona” associated with the nanoparticle [5,6]. Protein corona formation can be viewed as an interaction between nanoparticles and proteins [7–9]. This surface “biotransformation” of nanomaterials modulates their overall pharmacological and toxicological properties and their potential therapeutic or diagnostic functions in a rather unpredictable manner [10–12]. A thorough understanding of the composition of the protein corona and its interactions with nanoparticles is crucial for evaluating their biological effects and potential applications in biomedicine [13,14]. The protein corona composition reflects the relative protein abundance (RPA), which accounts for the total proteins in the corona, and is an important parameter for describing the protein corona [15]. Currently, most studies have employed liquid chromatography–tandem mass spectrometry (LC-MS/MS)-based proteomics techniques for the qualitative and quantitative characterization of the protein corona [16–21]. It is expensive and time consuming to perform the compositional analysis of protein corona using traditional experimental techniques. Therefore, artificial intelligence has been used to build predictive models to rapidly characterize the protein adsorption behavior and corona formation [22–25]. The implementation of standardized protocols for the methodology and analysis of protein corona can generate many multi-omics-based datasets. These datasets can be used for artificial intelligence to predict the formation of a protein corona on different nanoparticles [23].

To date, numerous factors (e.g., physicochemical properties of nanoparticles, incubation, and isolation conditions) have been shown to influence the biological response and composition of the protein corona [26–32]. Machine learning algorithms are well suited for extracting crucial information from these factors, enabling accurate prediction of the protein corona composition. The application of machine learning has the potential to enhance the scope and depth of research on the protein corona [33]. Numerous successful instances have demonstrated the application of machine learning to accurately identify the composition of the protein corona. Ouassil et al. [34] utilized a random forest (RF) classifier trained on MS data to identify proteins adsorbed onto nanoparticles based on protein sequences alone, achieving an accuracy of 78% and precision of 70%. Findlay et al. [22] supported the development of a protein corona prediction model using an RF classifier to correlate proteins, nanoparticles, and solution conditions with protein corona formation, achieving an area under the receiver operating characteristic curve (AUROC) of 0.83 and an F1 score of 0.81. Additionally, Ban et al. [35] used an RF regressor to accurately predict the RPA in the protein corona. Yu et al. [36] used an RF to accurately predict the lung immune response and burden in response to nanoparticles. These studies demonstrate the important potential of machine learning in the field of protein corona-related predictions, thereby facilitating protein corona-related research.

Unlike most of the previous studies [22–25] that used a single machine learning algorithm for simple prediction of the RPA of multiple proteins on the protein corona, the aim of this study was to employ machine learning models to comprehensively predict the RPA of multiple proteins on the protein corona, utilizing both classification and regression tasks to evaluate the performance of 6 different algorithms. Specifically, we aimed to determine whether multiple proteins were adsorbed onto the nanoparticle surface and to predict the specific RPA values, with a focus on elucidating the reasons behind the performance differences among the top-performing models through SHapley Additive exPlanations (SHAP) [37] analysis, which has not been done in previous studies. This is the inaugural study to utilize machine learning to generate comprehensive, interpretable predictions regarding the RPA of multiple proteins on protein corona. The service for the prediction of RPA is available at http://www.bioai-lab.com/PC_ML.

## Results

**Fig. 1.**
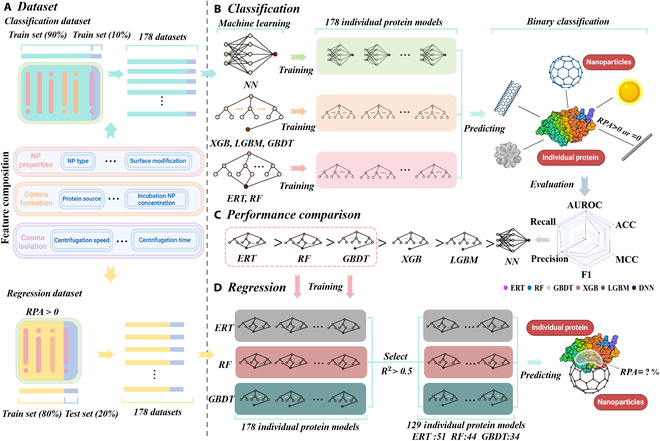
The overall experimental flow framework diagram. (A) The dataset used for the experiments contains a variety of features, the detailed names of which are given in Fig. S3. The dataset is divided for both the classification task and the regression task. (B) For classification prediction, 6 machine learning algorithms, namely, ERT, RF, XGB, LGBM, GBDT, and NN, are used to train in order to predict whether or not the RPA is greater than 0. (C) Evaluating the performance of the 6 types of machine learning in classification prediction thus selects the top 3 machine learning algorithms with better performance for the next step of regression prediction. (D) Regression prediction, using the selected ERT, RF, and GBDT to predict the specific value of RPA and selecting the model based on the evaluation metric *R*^2^.

In this study, the experiment was divided into 2 tasks: a binary classification prediction task, which predicted whether a protein was adsorbed on the nanoparticles, and a regression prediction task, which predicted the specific amount of a particular protein adsorbed on the protein corona. The experimental procedure is illustrated in Fig. 1.

### Binary classification prediction

**Fig. 2.**
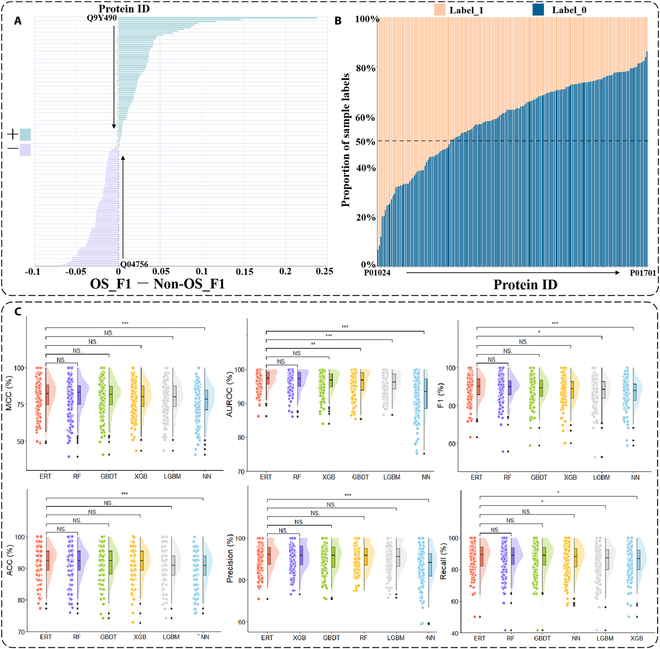
Demonstration of the effect of oversampling on ERT, distribution of samples in each dataset, and performance of 6 machine learning algorithm (ERT, RF, GBDT, XGB, LGBM, and NN) on different evaluation metrics in the classification task. (A) Value of the F1 metric on the test set for the models (ERT) using oversampling minus the value of the F1 metric on the test set for the models not using oversampling (OS_F1 − Non-OS_F1) on the different individual protein models. + represents that oversampling improves model performance, and − represents that oversampling reduces model performance. See Fig. S4 for complete details. (B) Percentage of different label values (0 or 1) in the 178 individual protein datasets. (C) Performance of the baseline model for the 6 machine learning algorithms on various evaluation metrics. Meanwhile, a *t* test was performed to compare the performance of ERT to that of the other algorithms on various evaluation metrics, with NS standing for no significant difference, and the higher the number of ⋆, the more significant the difference.

The dataset used for the experiment was taken from Ban et al. [35]. It comprised 178 individual proteins with 652 shared data points. Shared data points means that the feature data of these 178 individual proteins are the same, but the individual proteins are different and the trained labels are inconsistent. The feature data here mean the information data of the protein corona, which can be understood as the different proteins with different RPA (label) on the same protein corona. Consequently, the initial step in the experimental process involved dividing the training and test datasets, which was performed at a ratio of 9:1 for the 178 proteins. Simultaneously, the ratios of positive to negative samples in the training and test sets were maintained. Demonstration of the effect of oversampling on extremely randomized tree (ERT), distribution of samples in each dataset, and performance of each machine learning algorithm on different evaluation metrics in the classification task are shown in Fig. 2.

#### Model training and evaluation

Prior to training the model, the dataset was preprocessed using normalization, specifically max-min normalization. A normalization operation was employed to mitigate the negative impact of large differences in the data distribution on model training [38]. Following the normalization operation, 6 machine learning algorithms, namely, ERT [39], RF [40,41], gradient boosting decision tree (GBDT) [42], XGBoost (XGB) [43], Lightgbm (LGBM) [44], and neural networks (NN) [45–47], were employed to train the model, resulting in the generation of 178 baseline models for each machine learning algorithm. Each machine learning algorithm was comprehensively evaluated using 6 evaluation metrics: AUROC, Recall, Precision, F1, Matthews correlation coefficient (MCC), and Accuracy (ACC). The average performance of the 178 baseline models of each machine learning algorithm for each evaluation metric was used to assess the performance of each machine learning algorithm. As shown in Table 1, ERT exhibited the highest performance across all the evaluation metrics, indicating a remarkable advantage over the other 5 machine learning algorithms. Conversely, NN performed the least favorably in most evaluation metrics. The average performance on the validation sets is shown in Table S5. To further illustrate the differences in the predictions of the individual machine learning algorithms, we visualized the performance of the 178 baseline models of each machine learning algorithm, as shown in Fig. 2C. As illustrated in Fig. 2C, the performance results of the ERT exhibited greater compactness in the majority of evaluation metrics, with a relatively limited number of outliers. This indicates that ERT exhibited superior overall learning ability on a dataset of 178 individual proteins. A *t* test was employed to assess the discrepancy in performance between the ERT and other machine learning algorithms across all assessment metrics. There was a notable distinction between the ERT and NN in terms of AUROC, MCC, F1, ACC, and Precision. Thus, we can tentatively hypothesize that the prediction mode and learning focus of the ERT and NN may differ, which may explain the observed differences in performance. We will explain the learning differences between the ERT and NN through an interpretable analysis in a subsequent study. The figure also shows that ERT did not differ from the other traditional machine learning algorithms (RF and GBDT) in all 6 evaluation metrics, which informs our model selection for the next regression prediction task.

**Table 1.** Average performance of 6 machine learning algorithms on test sets of 178 independent proteins. Bold values indicate the best results.

	AUROC↑	Recall↑	Precision↑	F1↑	MCC↑	ACC↑
ERT	**0.969**	**0.877**	**0.915**	**0.893**	**0.811**	**0.919**
RF	0.967	0.875	0.910	0.889	0.804	0.916
GBDT	0.961	0.873	0.910	0.888	0.803	0.916
XGB	0.964	0.854	0.915	0.881	0.797	0.913
LGBM	0.959	0.855	0.908	0.877	0.785	0.909
NN	0.921	0.857	0.876	0.862	0.765	0.900

At the same time, in order to exclude the effect of the simplicity of the NN architecture we used (2-layer), we trained the NN with 3, 4, and 5 layers as well as the long short-term memory (LSTM) [48,49], and a comparison of their performance is shown in Table S4. It can be seen that the overall performance of the NN with 2-layer architecture is optimal, and the overall performance of the NN deteriorates as this number of layers increases, while the performance of the LSTM is unsatisfactory. Second, we find that as the architecture becomes more complex, the error in model interpretability becomes larger, so to ensure the accuracy of interpretability, we use the simpler 2-layer architecture of NN.

#### Oversampling

The preceding experimental results led to the selection of ERT as the final prediction model. The selection of hyperparameters for ERT and the tuning range are shown in Table S1. To enhance the performance of the 178 baseline ERT models, the sample label distribution on the 178 datasets was analyzed, and the results are presented in Fig. 2B. Figure 2B depicts the percentage of different label values (0 or 1) in the 178 individual protein datasets. The Fig. 2B evidently shows that some datasets exhibited a considerable proportion of positive samples, exceeding 0.5, whereas others exhibited a relatively small proportion of positive samples, falling below 0.5. Hence, the distribution of samples in the dataset of some individual proteins was uneven, which may result in the model failing to sufficiently learn the labels that account for a smaller proportion of samples. To address this issue, an oversampling strategy was employed to reconstruct the training set for each dataset. The resulting oversampled training set was used to retrain the model. As shown in Table 2, for 178 independent proteins, the average performance of ERT after oversampling has improved compared to before oversampling. Table 2 reveals that the model after oversampling exhibited a more pronounced enhancement in recall (nearly 2%), a marginal improvement in AUROC, F1, and MCC, a slight decline of 1.3% in precision, and a negligible reduction in ACC. As these values represent the average of 178 baseline model performances, the increase in recall and decrease in precision demonstrate that oversampling markedly enhanced the capacity of ERT to identify positive samples while concurrently reducing its ability to recognize negative samples. Concurrently, given the specificity between the labels of the 178 datasets, oversampling was not a suitable approach for all baseline models. Therefore, here, we employed the F1 score as an evaluation metric to distinguish between the positive and negative effects of oversampling on the baseline model, as shown in Fig. 2A. It can be seen that oversampling did not bring a positive gain effect on all individual protein models, but rather a negative gain effect on some of the models. Finally, an oversampling operation is performed on datasets that exhibit positive gains from oversampling.

**Table 2.** Average performance of ERT after oversampling versus before on 178 independent proteins. Bold values indicate the best results.

	AUROC↑	Recall↑	Precision↑	F1↑	MCC↑	ACC↑
Before oversampling	0.969	0.877	**0.915**	0.893	0.811	**0.919**
After oversampling	**0.970**	**0.895**	0.902	**0.896**	**0.818**	**0.919**

There is also an undersampling method that can be used to deal with data imbalance. In contrast to oversampling, undersampling balances the dataset based on a smaller number of samples. As shown in Fig. 2B, some protein datasets have a very small number of samples (e.g., Protein ID: P01024), and if undersampling is used, the total number of samples in these datasets is less than 100, and the small number of samples will lead to insufficient model training. Of course, we also verify our conjecture by experiment. The average performance of ERT after oversampling and undersampling versus before on 178 independent proteins is shown in Table S2. It can be seen that undersampling reduces the overall effect, although undersampling slightly improves the recall, but oversampling can bring better result. As shown in Fig. S5, individual protein models using oversampling negative gain perform better than without oversampling. Then, we also conducted experiments using the sample weight change method. A comparison of the average performance on these individual proteins using the undersampling and sample weight change methods with and without these methods is shown in Table S3. It can be seen that these methods did not improve the performance of these individual protein models overall. Therefore, for these individual protein models that already perform well, we do not use oversampling or undersampling.

#### ERT versus NN

Our initial hypothesis was that the discrepancy between the NN and ERT was due to the divergence in their learning focus. To validate this hypothesis, we conducted an interpretable analysis of the ERT and NN. Based on the results of previous experiments, we obtained 178 baseline ERT models. Because of the differences between each baseline model, an interpretable analysis of all 178 baseline models was relatively tedious, complex, and repetitive. Therefore, we selected a “representative” from these 178 baseline models. A correlation analysis of the feature importance of the 178 baseline models of ERT was conducted, and the “representative” baseline model was selected as the one with the highest mean value of correlation with the other 177 baseline models. The correlation heatmap for the 178 baseline models is shown in Fig. S2. We obtained a “representative” baseline model, corresponding to the protein ID “P02766”. Subsequently, the ERT and NN baseline models with protein ID “P02766” were analyzed for interpretability.

**Fig. 3.**
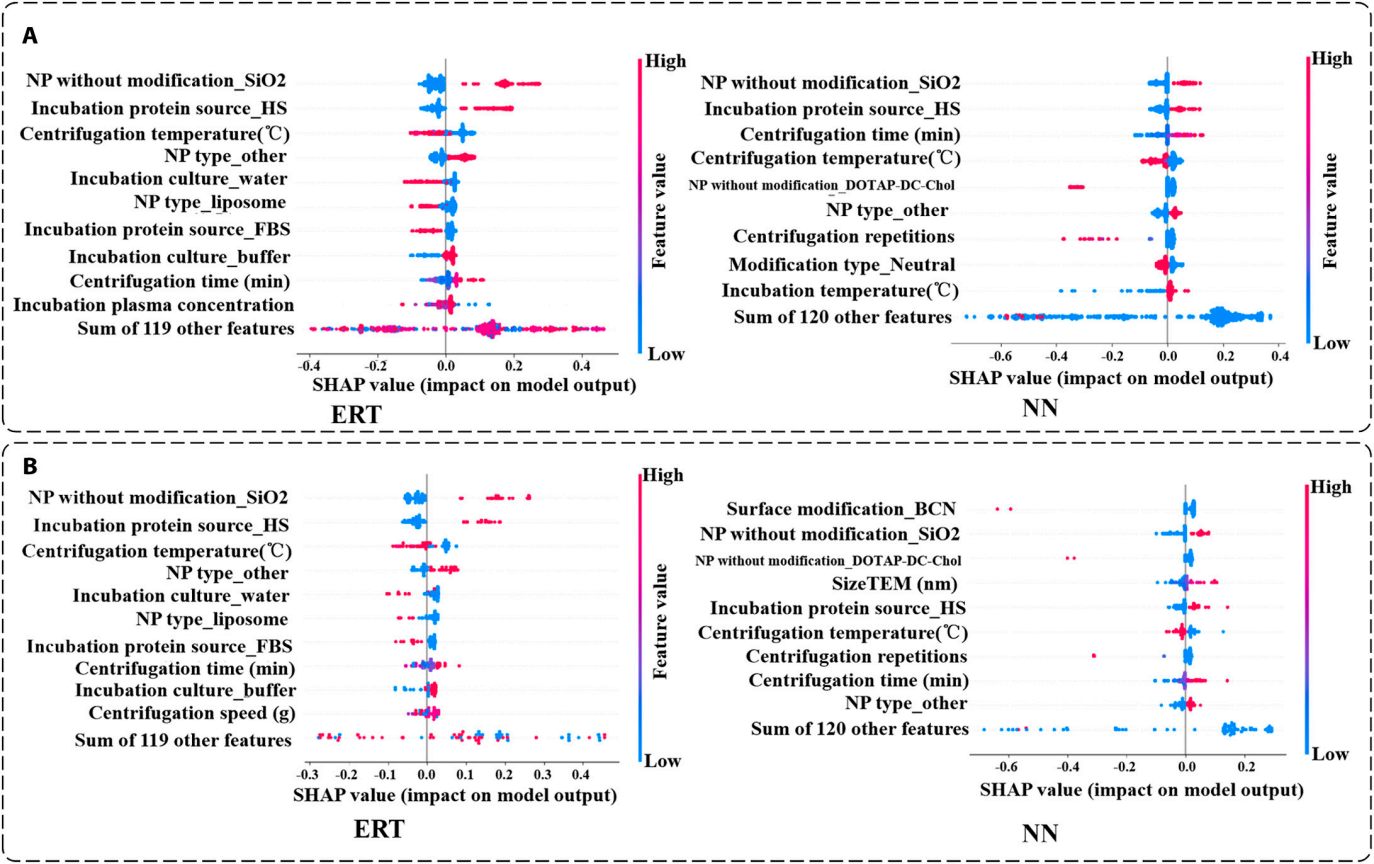
Differences in learning patterns between ERT and NN through interpretable analyses are demonstrated. (A) Analysis of the contribution of the respective features of ERT versus NN on the training set. (B) Analysis of the contribution of the respective features of ERT versus NN on the test set.

A comprehensive interpretability analysis of the ERT and NN was performed using the SHAP method. First, we drew a feature contribution histogram using the shap values, as shown in Fig. S6. The feature “NP without modification” showed the largest contribution to both ERT and NN. However, the feature importance distribution of the other features shows a clear difference between ERT and NN. To facilitate a more detailed analysis, we present the beeswarm plots of ERT and NN on the training and testing sets in Fig. 3A and B, respectively. There was a clear distinction between the primary feature contributions of ERT and NN, both on the training set and the test set. This observation corroborated our initial hypothesis. Furthermore, it is evident that the ERT rankings of the top 10 feature contributions were largely consistent across the training and test sets. Conversely, it is evident that the distributions of the top 10 feature contributions of the NN on the training and test sets were disparate.

**Fig. 4.**
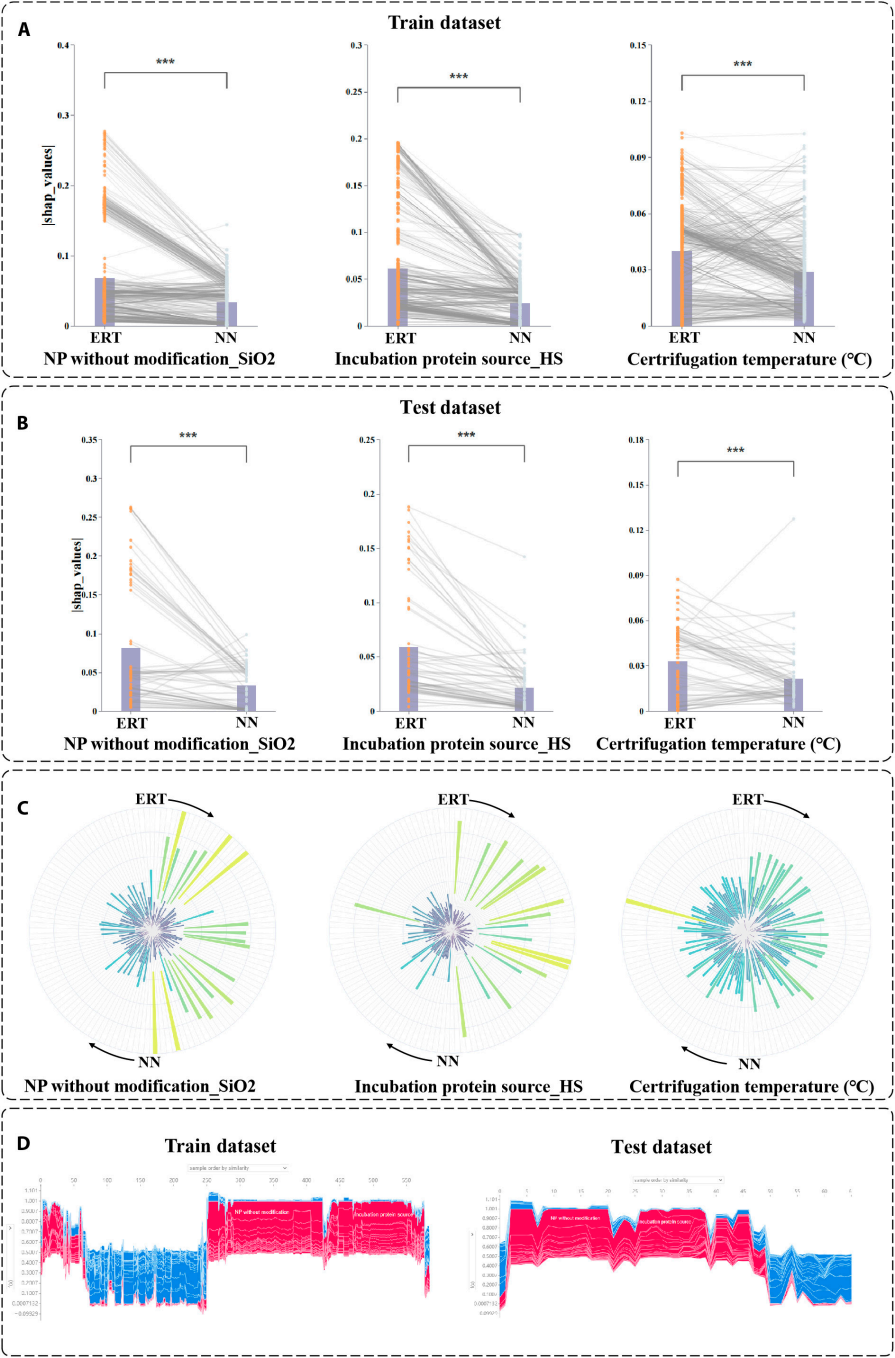
Visualization of the shap values to compare the differences in learning of main features (NP without modification_SiO2, Incubation protein source_HS, and Centrifugation temperature) by ERT and NN. (A) Visualization of the differences in performance of main features on ERT and NN in terms of shap values on the training set and perform a *t* test, and the higher the number of ⋆, the more significant the difference. (B) Visualization of the difference in performance of main features on ERT and NN in terms of shap values on the test set. (C) Relative magnitude of the shap values on ERT and NN for the main features on the test set. (D) Explainable force plots of ERT on the training and test sets with respect to all samples.

In order to more comprehensively understand the learning differences between ERT and NN, we selected the 3 features that perform optimally on ERT: “NP without modification_SiO2”, “Incubation protein source_HS”, and “Centrifugation temperature”. We then visualized the shap values of these 3 features of the ERT and NN on the training and test sets, as shown in Fig. 4A and B. In this analysis, the absolute shap values for the study were obtained, and a *t* test was employed to assess the disparities exhibited by the gap values of the 3 features on ERT and NN. The shap values of the 3 features in the ERT and NN exhibited a notable discrepancy in both the training and test sets. The shap values of the feature “NP without modification_SiO2” on ERT were considerably higher than those of NN. With regard to the feature “Incubation protein source_HS”, its performance is comparable to that of the feature “NP without modification_SiO2”. Similarly, there is a notable discrepancy between ERT and NN with regard to the feature “Centrifugation temperature”. Furthermore, we conducted a test set analysis, as illustrated in Fig. 4C. The left side of the circle in the figure indicates the relative magnitude of the shap values on the ERT, and the right side of the circle shows the relative magnitude of the shap values on the NN. The position of the same sample on the ERT and NN was symmetric about the center of the circle.

Finally, we demonstrated the impact of the shape values of each feature of the ERT on the model prediction results for the training and test sets using force plots, as illustrated in Fig. 4D. Both on the test set and the training set, the features “NP without modification” and “Incubation protein source” exert a profound influence on the model’s decision-making process.

### Regression prediction

Once an accurate prediction of whether a protein has been adsorbed on a nanoparticle has been made, the subsequent step is to predict the relative amount of protein adsorbed on the nanoparticle, that is, the specific value of the RPA. This is a regression prediction problem. In addition, machine learning was employed for prediction purposes, with 3 traditional machine learning algorithms—ERT, RF, and GBDT.

#### Data preprocessing

A total of 178 datasets were preprocessed prior to training. In the regression prediction task, our data should be restricted to an RPA value greater than 0. Consequently, 178 datasets were filtered, and the training and test sets were divided in a ratio of 8:2. Following the division of the dataset, a max-min normalization operation was applied to prevent the difficulty of model training caused by large differences in data distribution.

#### Model training and evaluation

For the regression task, we identified 3 models that performed better on the protein corona information dataset: ERT, RF, and GBDT. First, we employed 10-fold cross-validation with a random grid search to train the baseline model and search for optimal hyperparameters.

Similarly, 178 baseline regression models were obtained for the 3 machine learning algorithms. An initial evaluation of these baseline models was performed using coefficient of determination (*R*^2^) and root mean square error (RMSE), and the average performance of ERT, RF, and GBDT on test sets of 178 independent proteins is shown in Table 3. The average performance on the validation sets is shown in Table S6. As shown in Table 3, the RF baseline models exhibited the highest performance on the *R*^2^ metric, whereas the ERT models demonstrated the highest performance on the RMSE metric. Since our objective was to identify the baseline model that performed optimally for a given dataset, we selected the model that performed best against a particular dataset based on the *R*^2^ metrics. Following the selection process, ERT performed the best on 66 datasets, RF performed the best on 61 datasets, and GBDT performed the best on 51 datasets. However, among these selected baseline models, some exhibited suboptimal performances (*R*^2^ < 0.5). This may be attributed to the fact that the datasets themselves were not optimal for regression prediction, leading to the inferior performance of these baseline models, which tend to yield biased prediction results. Consequently, baseline models with *R*^2^ value below 0.5 were excluded. Finally, for the ERT, 51 baseline models with *R*^2^ value greater than 0.5 were retained. The same procedure was applied to RF and GBDT, resulting in the retention of 61 baseline models for RF and 51 baseline models for GBDT. In summary, for the 178 datasets corresponding to the optimal baseline models, 129 better-performing (*R*^2^ > 0.5) baseline models were retained.

**Table 3.** Average performance of ERT, RF, and GBDT on test sets of 178 independent proteins in regression prediction. Bolding indicates the best results.

	RMSE ↓	*R*^2^ ↑
ERT	**0.758**	0.503
RF	0.760	**0.534**
GBDT	0.760	0.489

#### Interpretable analysis

**Fig. 5.**
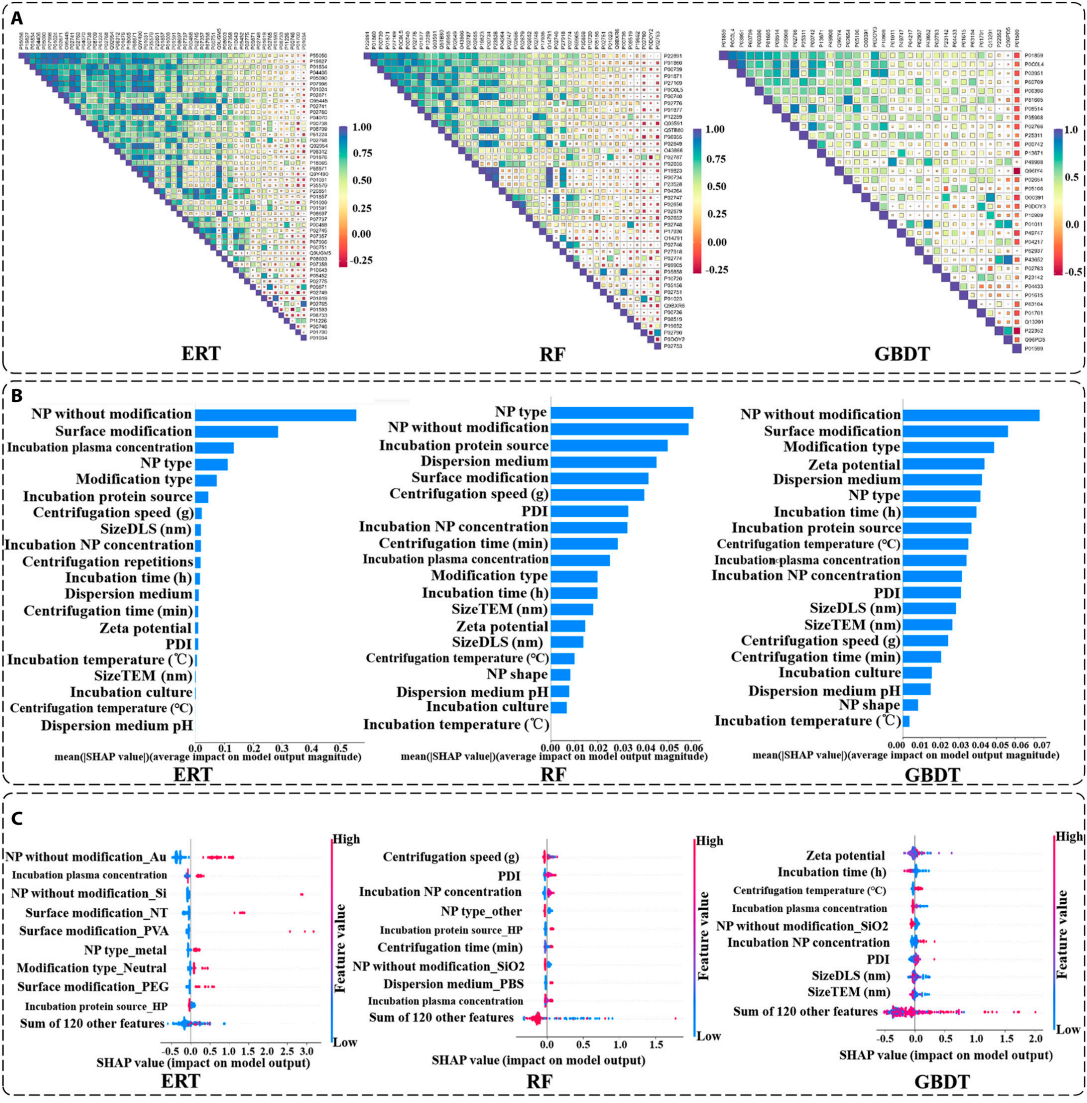
Interpretable analysis of regression models. (A) Heatmaps of the correlation between the feature importance of the respective baseline models for ERT, RF, and GBDT. (B) Preliminary assessment of the feature importance of ERT, RF, and GBDT. (C) Analysis of the contribution of the respective features of ERT, RF, and GBDT.

In order to gain further insights into the learning patterns of these 3 distinct machine learning models, an interpretable analysis was conducted on the 3 aforementioned machine learning models. As with the classification model interpretability analysis in the “ERT versus NN” section, we begin by selecting a “representative” model for each of the 3 machine learning algorithms. The methodology for selecting the “representative” model was consistent with that used in the “ERT versus NN” section. The correlation heatmap of the baseline models for ERT, RF, and GBDT is shown in Fig. 5A. The “representative” models for ERT, RF, and GBDT corresponded to the protein IDs “P55056”, “P22891”, and “P01859”, respectively.

First, we conducted a statistical analysis of the importance of each feature of the 3 “representative” baseline models, as shown in Fig. 5B. Typically, the importance of the performance of each feature in ERT, RF, and GBDT is remarkably different. To further illustrate the performance of each feature in these 3 distinct machine learning models, we created their respective beeswarm plots, as shown in Fig. 5C. The beeswarm plots allow for a more detailed visualization of the learning of each feature by ERT, RF, and GBDT.

**Fig. 6.**
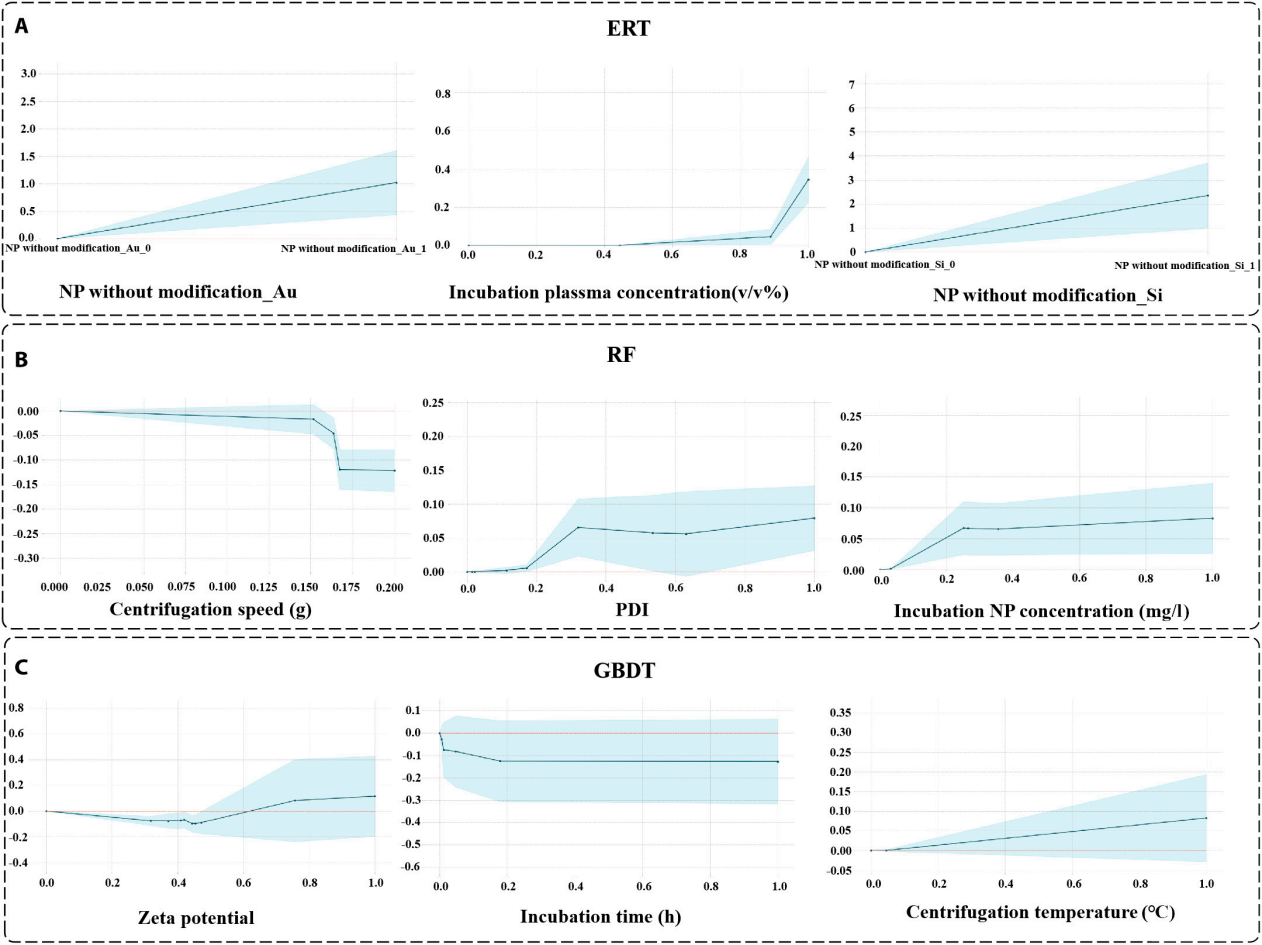
Partial dependency analysis of respective important features on ERT, RF, and GBDT. (A) ERT. (B) RF. (C) GBDT.

**Fig. 7.**
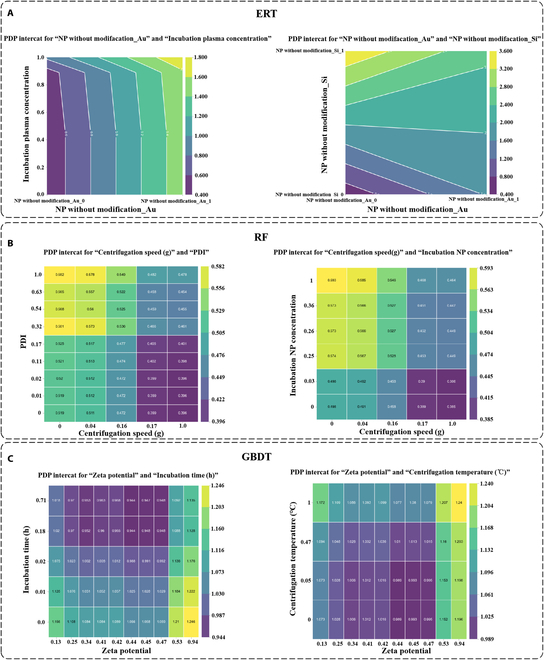
Impact of the interactions between respective main features on model outputs of ERT, RF, and GBDT. (A) ERT. (B) RF. (C) GBDT.

For ERT, the top 3 performing features were “NP without modifica_Au”, “Incubation plasma concentration”, and “NP without modifica_Si”. The feature “NP without modification_Au” is a one-dimensional representation of the result of encoding the feature “NP without modification” using the one-hot encoding. The value of “NP without modification_Au” indicates whether the nanoparticle is a gold nanoparticle, taking the value of 0 or 1. When the nanomaterial was a gold nanoparticle, it exhibited a positive contribution to the model. We then examined “Incubation plasma concentration”, a numerical feature. As illustrated in Fig. 5C, the smaller the value of “Incubation plasma concentration”, the more it contributed negatively to the model. Finally, the feature “NP without modification_Si” was also one-dimensional in the result of the one-hot encoding of the feature “NP without modification”. When the nanoparticle material was silicon, it exerted a substantial positive influence on the model. Subsequently, we examined the correlation between the values of these features and the final results of the model output through the partial dependency plots of these 3 features, as shown in Fig. 6A. As illustrated in the figure, when the value of “NP without modification_Au” transitioned from 0 to 1, the model’s overall prediction on the samples exhibited a notable increase, aligning with the findings observed in the beeswarm plot. Similarly, when the “Incubation plasma concentration” value ascended from 0 to 1, the model’s overall prediction also demonstrated a consistent upward trend. The value of “Incubation plasma concentration” was constrained to a range of 0 to 1 due to the normalization operation. Finally, when the value of the feature “NP without modification_Si” was changed from 0 to 1, the prediction result of the model also tended to become larger. The alteration of the “NP without modification_Si” feature yielded a larger output than that of the “NP without modification_Au” feature, which was also consistent with the results displayed in the beeswarm plot. Subsequently, we examined the interrelationship between the interaction between the “NP without modification_Au” feature and the other 2 features, as well as the output of the model, as illustrated in Fig. 7A. When the value of “NP without modification_Au” was 1, the larger the value of “Incubation plasma concentration”, the larger the output result given by the model. When the value of “NP without modification_Au” was 0, the model showed lower output results. When the value of “NP without modification_Au” was 0 and the value of “NP without modification_Si” was 1, the model showed larger predictions. When the value of “NP without modification_Au” was 0 and the value of “NP without modification_Si” was also 0, the model showed smaller predictions. The aforementioned findings show the interpretable analysis for the “representative” baseline model of ERT, and the same approach was used for RF and GBDT.

For RF, the impact of the values of the individual features on the model predictions can be observed in the beeswarm plot shown in Fig. 5C. Similarly, the 3 most influential features, in terms of importance, are identified as “Centrifugation speed” “PDI” and “Incubation NP concentration”. From the partial dependence plots (PDPs) of the RF features in Fig. 6B, it can be observed that when the “Centrifugal speed” is higher, the overall prediction result given by the model tends to decrease in size. Conversely, when the values of the 2 features “PDI” and “Incubation NP concentration” are larger, the size of the overall prediction results given by the model tends to increase. The performance of these 3 features in the partial dependency plots was generally consistent with the results shown in the beeswarm plot of RF. Figure 7B illustrates the effect of the interaction between the feature “Centrifugation speed” and the other 2 features on the model’s prediction. It can be seen that when the value of “Centrifugation speed” is smaller and the value of “PDI” is larger, the model produces larger predictions. Conversely, when the value of “Centrifugation speed” is larger and the value of “PDI” is smaller, the model produces smaller predictions. Similarly, when the value of “Centrifugation speed” is smaller and the value of “Incubation NP concentration” is larger, the model produces larger predictions. Conversely, when the value of “Centrifugation speed” is larger and the value of “Incubation NP concentration” is smaller, the model produces smaller predictions.

The 3 most important features in the “representative” baseline model of GBDT, as identified according to the beeswarm plot of GBDT in Fig. 5C, were “Zeta potential”, “Incubation time”, and “Centrifugation temperature”. Similarly, the partial dependency plots of the GBDT features in Fig. 6C indicate that the overall prediction by the model decreased and then increased as the “Zeta potential” increased. As the value of “Incubation time” increases, the overall prediction result of the model first decreased and then stabilized. When the value of “Centrifugation temperature” was larger, the overall prediction result of the model showed an upward trend. Figure 7C illustrates the impact of the interaction between the “Zeta potential” feature and the other 2 features on the model predictions. When the “Zeta potential” value was higher and the “Incubation time” value was lower, the model generated larger prediction outcomes. Conversely, when the “Incubation time” value was higher, the model generated relatively smaller prediction outcomes. In contrast, the model yielded larger predictions when the values of “Zeta potential” and “Centrifugation temperature” were high. Conversely, when the “Centrifugation temperature” was lower, the model generated relatively smaller predictions.

## Discussion

In this study, we employed machine learning methods to comprehensively predict the RPA of multiple proteins on the protein corona. For the classification task, we compared the performance of 6 machine learning algorithms and selected the best performing ERT and the worst performing NN. We identified the reasons for the difference in performance between ERT and NN through interpretable analysis. First, it is understood that the feature “NP without modification” contributes importantly to both ERT and NN. However, during the learning process, ERT successfully transferred the experience acquired from the training set to the test set, which NN does not do. Meanwhile, ERT learns more fully for this feature. We then analyzed the architecture of the NN and found that the more complex the architecture, the more likely it is to suffer from overfitting, which is one of the reasons why the NN performs poorly on the protein corona datasets. Finally, in order to further improve the performance of ERT’s performance, we experimented with oversampling, undersampling, and adjusting sample weight methods and found that the undersampling and adjusting weight methods are not as effective as oversampling and also that oversampling does not apply to all datasets, on which ERT itself has good performance. These analyses provide valuable suggestions for the application of machine learning in the correlation prediction of protein corona. Important features related to the RPA of proteins on protein corona such as “NP without modification” and “Incubation protein source” were also mined, which can be used as a reference for the initial design of protein corona.

For the regression task, the feature “NP without modification” also shows a large contribution. A comprehensive interpretable analysis was performed holistically and locally, focusing on the shap values and partial dependencies of the features. The learning patterns and priorities of ERT, RF, and GBDT on the protein corona datasets were elucidated. Concurrently, the impact of the values of crucial features and the interconnections between the principal features on the model outputs was evaluated, providing a foundation for further comprehension of the learning processes of the models and a framework for further enhancement of their performance. Meanwhile, this provides a deeper reference for designing desirable and safe nanoparticles for nanomedicine, biosensing, organ targeting, and other applications.

The classification prediction task and the regression prediction task in our experiments are 2 separate tasks, and the models from the 2 tasks are trained separately. From this level, no error aggregation occurs. So even if the classification task gives any results it will not affect the results given by the regression task. One of the purposes of doing them separately is to make the regression task training more efficient because we filter the data for the regression task to ensure that the labeling of each piece of data is meaningful, i.e., RPA > 0. This is because if RPA = 0, the protein is not adsorbed on the nanoparticles, and then predicting the amount of adsorption of that protein on the nanoparticles is meaningless. Second, the classification model will give preliminary suggestions, which can greatly reduce the design cost of protein coronas. Our tools (http://www.bioai-lab.com/PC_ML) can provide both of these functions.

Also, due to the high duplication of the dataset used, 178 proteins sharing 652 data points, the form of data used is not nanoprotein pairs, thus requiring modeling of each of the 178 proteins individually. Due to the specificity of the individual protein RPA values and the high data duplication, it is difficult to achieve accurate prediction of the RPA values of all individual proteins. This is further exemplified in the regression prediction task, where the available valid data are different for different proteins, and some proteins correspond to relatively small datasets, which may lead to under-training of the model.

However, there are some limitations as well. We have to model individual proteins separately, and a baseline model can only predict for one protein, which is inconvenient. To address this limitation, it is possible to consider fusing features of proteins in the training of the model so that the model can recognize different proteins as a way to enhance the generalization ability of the model with respect to proteins. Meanwhile, extracting features about proteins may enrich the feature representation of protein corona more, which is beneficial for further mining important features about protein corona-related prediction.

## Materials and Methods

In this study, we constructed machine learning models to predict the RPA of a protein corona. Initially, a binary prediction model was trained to determine whether the RPA was greater than zero. Subsequently, for instances where the RPA was greater than zero, we trained a regression model to predict the specific value of the RPA. By combining these 2 prediction tasks, we were able to achieve a comprehensive and adequate RPA prediction.

### Dataset

The dataset used for the experiments was derived from a study conducted by Ban et al. [35]. Through a comprehensive literature search, Ban et al. extracted 652 data points pertaining to the protein corona of nanoparticles. These data points were then used to estimate the RPAs of 178 independent proteins, employing normalized spectral counts (NSCs) of the proteins in the protein corona. The aforementioned data were subsequently employed to construct corresponding machine learning models for 178 independent proteins to predict their respective RPAs.

### Features

A total of 652 data points comprised 21 important features related to the NP properties, corona formation, and corona isolation. A total of 21 important factors consisted of 8 qualitative factors (nanoparticle type and shape, those without modification, surface modification, modification type, dispersion medium, incubation plasma source, and incubation culture) and 13 quantitative factors [size measured by transmission electron microscopy (size TEM) and dynamic light scattering (size DLS), dispersion medium pH, zeta potential, polydispersity index, incubation plasma concentration, incubation nanoparticle concentration, incubation time, incubation temperature, centrifugation speed, centrifugation time, and centrifugation repetitions]. For these qualitative factors, 40 types of nanoparticles were noted without modification including carbonaceous (e.g., multi-walled carbon nanotubes and single-walled carbon nanotubes), metallic (e.g., Ag and Au), nonmetallic (e.g., SiO2 and Si), and liposomal (e.g., cholesterolphosphatidylcholine and thiolated amino-poly[ethylene glycol]) types. A total of 50 types of surface modifications, including anionic (e.g., *N*-acetyl-l-cysteine and thiolated l-asparagine), cationic (e.g., 11-amino-1-undecanethiol and hexadecyltrimethylammonium bromide), and neutral (e.g., carboxymethyl-poly[ethylene glycol]-thiol and bicyclononyne) modifications, were recorded. The distribution of the nanoparticle properties is shown in Fig. S1. To enable the machine learning model to learn these 8 qualitative factors, we encoded them into a numerical form using one-hot encoding. We hypothesized that the 13 quantitative factors should remain unaltered and used directly as features. After processing, we split the encoded qualitative factor features into quantitative features. Finally, a 129-dimensional feature representation was obtained for each sample. To mitigate the adverse effects of differences in data distribution during model training, we preprocessed the data using the max-min normalization method, whereby individual feature values were compressed to a range between 0 and 1. The max-min normalization operation was characterized via the following equation:

$$x_{\mathrm{norm}}=\frac{x-x_{\min}}{x_{\max}-x_{\min}}$$ (1)

where *x* denotes the original feature, *x_max_* is the maximum value of the feature, *x_min_* is the minimum value of the feature, and *x_norm_* is the feature after normalization. In our experiments, we directly used these preprocessed features to train our models and perform the corresponding interpretable analyses.

### Binary classification prediction task

#### Machine learning algorithms

In this study, an RPA of a protein on the protein corona greater than 0 indicated that the corresponding protein was attached to the surface of the nanoparticle, whereas an RPA equal to zero signified that the nanoparticle surface did not adsorb the corresponding protein. Therefore, we initially focused on the question of adsorption, which was transformed into a binary classification prediction task using 6 different machine learning models to predict whether the RPA values of individual proteins in the protein corona were greater than 0. The 6 selected machine learning models included 5 traditional machine learning models, ERT, RF, GBDT, XGB, and LGBM, as well as a neural network model, NN. First, we introduced the classic machine learning algorithm, RF, which is an extended variant of the bagging integration algorithm. RF further introduced randomized attribute selection during the training process of the decision tree, using the decision tree as the base learner, to build bagging integration. Notably, a traditional decision tree selects an optimal attribute from the attribute set of the current node when selecting the attributes for division. In contrast, in the RF, for each node of the base decision tree, some attributes are randomly selected as a subset from the attribute set of the node, and then an optimal attribute from this subset is selected for division. For the classification problem, the RF prediction formula can be expressed as:

$$\hat{y}=\underset{y}{\arg\;\max}\sum_{k=1}^{K}I\left(h_{k}\left(x,\Theta_{k}\right)==y\right)$$ (2)

where $\hat{y}$ represents the final classification result, *y* represents the classification label (for a binary classification problem, it can be defined as *y* ∈ {0, 1}), *K* represents the number of base classifiers in RF, *I*(∘) represents the indicator function, *h_k_*(∘) represents the *k*th base classifier in RF, *x* represents the input data, and Θ*_k_* represents the random attribute subset of the *k*th base classifier.

ERT was then introduced, which is also a tree-based integration algorithm and a further extension of the RF. Compared with RF, ERT extends the concept of randomization even further by randomly selecting a portion of the features at each node, in addition to randomly selecting the splitting threshold, instead of searching for the optimal threshold. This additional randomization renders ERT more resistant to overfitting and facilitates faster training than RF. Similar to RF, ERT also requires the random selection of a subset of attributes. Hence, the prediction formula for ERT can be derived from Eq. 2 as an integrated algorithm.

GBDT is a representative boosting algorithm, and its modeling process is serial. Based on the results of the previous weak evaluator, a loss function was computed, and the loss of the current weak evaluator was used to adaptively influence the construction of the next weak evaluator. The integrated model outputs results that are influenced by all the weak evaluators. In particular, GBDT’s weak evaluators are regressors, regardless of whether they perform regression or classification tasks. Consequently, for classification tasks, the GBDT outputs specific classification results via sigmoid or softmax activation functions. Therefore, drawing on the boosting algorithm process, the binary classification prediction process of the GBDT can be characterized as follows:

$$H\left(x\right)=\sum_{t=1}^{T}\phi_{t}h_{t}\left(x\right)$$ (3)

$$p\left(\hat{y}=1|x\right)=\sigma \left(H\left(x\right)\right)$$ (4)

where *x* represents the sample data, *H*(*x*) represents the result of GBDT integration, *T* represents the number of weak evaluators, *ϕ_t_* represents the weight of the *t*th weak evaluator, *h_t_*(∘) represents the *t*th weak evaluator, *σ* is the sigmoid activation function when $p\left(\hat{y}=1|x\right)>0.5$, the prediction category is 1, and vice versa is 0. At the same time, the formula of *ϕ_t_* in the iterative process is:

$$\phi_{t}=\underset{\phi }{\arg\;\min}\sum_{i=1}^{M}L\left(y_{i},H_{t-1}\left(x_{i}\right)+\phi_{t}h_{t}\left(x_{i}\right)\right)$$ (5)

where *M* is the number of samples in the dataset and *L*(∘) is the loss function. Then, the model is iterated based on the solved weights *ϕ_t_*:

$$H_{t}\left(x\right)=H_{t-1}\left(x\right)+\eta \phi_{t}h_{t}\left(x\right)$$ (6)

where *η* is the learning rate. The GBDT performs relevant modeling and iterations based on the aforementioned process, integrating the results of all weak evaluators through the boosting method.

The XGB represents a novel generation of boosting algorithms derived from GBDT and is enhanced with a novel regularization strategy. In contrast to the GBDT, XGB incorporates this strategy, which is absent from the GBDT. In contrast, XGB employs an approximate greedy algorithm for the selection of split points, which is more efficient than the global optimal split-point search employed by the GBDT. XGB offers enhanced support for parallel computing, enabling the utilization of multithreading and distributed computing, which accelerates the training process. As a boosting algorithm, the XGB employs an iterative process similar to that of the GBDT. However, in contrast to the GBDT, the XGB does not compute the weights of the weak evaluator, resulting in the following output:

$$H\left(x\right)=\sum_{t=1}^{T}h_{t}\left(x\right)$$ (7)

Meanwhile, the *t*th iteration can be expressed as:

$$H_{t}\left(x\right)=H_{t-1}\left(x\right)+\eta h_{t}\left(x\right)$$ (8)

LGBM is a machine learning algorithm based on the GBDT. Compared to the traditional GBDT, the LGBM exhibits notable advantages in terms of training speed, memory usage, and model performance. The LGBM employs a histogram-based decision tree algorithm that discretizes continuous feature values into a limited number of buckets and then performs decision tree construction on each bucket. This approach reduces the complexity of the decision tree and consequently accelerates the training process. The LGBM employs a leaf-wise growth strategy, whereby the node with the greatest gain from the current leaf node is selected for splitting each time. This strategy enables the identification of a more optimal splitting point with greater efficiency than the level-wise growth strategy. Furthermore, the LGBM utilizes histogram-based gradient boosting to calculate the split-point gain, which avoids the need to recalculate the gradient information of all data in each iteration, thereby accelerating the calculation speed.

NNs, inspired by the principles of the biological nervous system, are computational models based on interconnected artificial neurons and are also referred to as nodes or units. They can learn and simulate complex nonlinear relationships, thus serving as powerful tools for addressing various machine learning and artificial intelligence tasks. NNs typically consist of multiple layers, including an input layer, one or more hidden layers, and an output layer. Each layer comprises multiple neurons, and each neuron is connected to all neurons in the subsequent layer. The strengths of these connections are represented by weights, and each neuron is associated with a bias term. To ensure interpretability through the application of SHAP, we constructed a simple single-hidden layer feed-forward network for training. The basic prediction process is outlined as follows.

$$X_{\text{hidden}}=\sigma \left(XW_{1}+b_{1}\right)$$ (9)

$$Y_{\text{prob}}=\text{Sigmoid}\left(X_{\text{hidden}}W_{2}+b_{2}\right)$$ (10)

where *X* denotes the input data, *W*_1_ denotes the first linear layer weight, *b*_1_ denotes the bias term of the first linear layer, *σ*(∘) denotes the ReLU activation function, *X_hidden_* denotes the data processed by the first linear layer, *W*_2_ denotes the second linear layer weight, *b*_2_ denotes the bias term of the second linear layer, and *Y_prob_* denotes the predicted probability of the final output of the model. We conducted a comparative analysis of the performance of 6 machine learning models on 178 independent protein datasets to identify the most effective model.

#### Oversampling, undersampling, and adjusting sample weights

For the classification prediction task, we analyzed the sample distribution of the 178 datasets used for the experiment as shown in figure. We found that most of the datasets have uneven distribution of positive and negative samples, so we used synthetic minority over-sampling technique (SMOTE) [50] to oversample the datasets. The principle of the SMOTE algorithm is to randomly select a nearest-neighbor sample *b* for the small number of samples *a* and then randomly select a point *c* as a new small number of samples of the class from the connecting line between *a* and *b*. SMOTE first applies the Euclidean distance to calculate the distance of all the data of the minority sample from the minority sample point a to obtain the *k* nearest neighbors (*k* nearest data to the sample point). Then set a sampling ratio based on the proportion of sample imbalance and determine the sampling multiplier based on the sampling ratio as *N*. For each small number of class samples *x*, a number of samples are randomly selected from the *k* nearest neighbors, and the nearest neighbors obtained for the sample *x* are *x_n_*. For each randomly selected nearest neighbor *x_n_*, a new sample is constructed according to the following equation:

$$x_{\mathrm{new}}=x+\mathrm{rand}\left(0,1\right)*\left(x_{n}-x\right)$$ (11)

where *rand*(0, 1) means to select a number randomly from 0 to 1. After the SMOTE operation, we used the oversampled dataset to retrain the machine learning model and evaluated the benefit from oversampling.

We also use undersampling [51] to deal with the data imbalance problem. In contrast to oversampling, undersampling balances the data by reducing the number of samples in the majority category, thus solving the situation where the number of samples in the majority category is much larger than the number of samples in the minority category in a classification problem.

Finally, we also used the method of adjusting the sample weights. In classification problems, when the number of samples in some categories is much less than others, the contribution of different categories can be balanced by adjusting the sample weights to avoid the model being overly biased toward the majority category, thus improving the generalization ability and accuracy of the model. In our experiments, we compared the advantages, disadvantages, and contributions of these 3 methods.

#### Interpretable analysis

In order to fully understand the decision-making process of the machine learning models, we performed an interpretable analysis of the models based on the SHAP approach. For each prediction sample, SHAP helps to understand how the model makes decisions by calculating the Shapley value of each feature and decomposing the predicted value of the model output into the contribution of each feature. Based on SHAP, the predicted value of the model *y* = *f*(*x*) can be obtained with the following equation:

$$f\left(x\right)=g\left(x^{\prime }\right)=\psi_{0}+\sum_{j=1}^{M}\psi_{j}x_{j}^{\prime }$$ (12)

where *g* is the concomitant model used for interpretation, $x_{j}^{\prime }$ indicates whether feature *ψ_j_* can be observed or not, and features that cannot be observed will not contribute to the interpretation. *M* is the number of input features, *ψ_j_* is the Shapley value of each feature, and *ψ*_0_ is the interpretation model constant, whose value is equal to the predicted mean value of all training samples. We performed a fully interpretable analysis of ERT and NN based on SHAP to compare the learning differences between these 2 different types of machine learning algorithms on protein corona information data, as well as to uncover important features related to RPA classification prediction.

### Regression prediction task

Another area of interest in this study was the amount of protein adsorbed onto the nanoparticles. The specific value of RPA reflects the relative amount of protein adsorbed onto the nanoparticles. Therefore, based on the previous step of the study, we used a machine learning modeling algorithm to predict the specific RPA value for each protein in the protein corona. This allowed us to comprehensively predict the RPA. The 3 most effective modeling algorithms, ERT, RF, and GBDT, as determined by their performance in the preceding research phase, were selected for further analysis. For the regression prediction task, the prediction formula for RF can be rewritten from Eq. 2 as:

$$\hat{y}=\frac{1}{K}\sum_{k=1}^{K}h_{k}\left(x,\Theta_{k}\right)$$ (13)

where $\hat{y}$ represents the final regression result, *K* represents the number of base regressors in RF, *h_k_*(∘) represents the *k*th base regressor in RF, *x* represents the input data, and Θ*_k_* represents the random attribute subset of the *k*th base regressor. The prediction process of the ERT regressor is referred to as the prediction process for the RF regressor. Because the weak evaluator of the GBDT is a regressor, regardless of whether it performs the regression or classification task, the prediction process of the GBDT regressor can be referred to in Eq. 3, and the GBDT regressor does not need to perform the operation described in Eq. 4.

These algorithms were employed to predict the specific RPA values of 178 individual proteins on the protein corona. The algorithms with the most favorable performances were identified. Similarly, an interpretable analysis was conducted to examine the differences among the 3 algorithms in their ability to predict the RPA values on protein corona. In addition to the SHAP-based interpretable approach, we also used the PDP [52] to analyze the impact of the main features on the model output, showing how the model predictions vary with individual features. We also analyzed the effect of combinations of major features on model output using PDP, which allows us to understand how the model handles the interaction of combinations of major features. With PDP, we further mined the impact of feature interactions on model predictions.

### Evaluation metrics

In this study, we initially sought to ascertain whether the RPA of protein corona was greater than zero, which represented a binary prediction. Subsequently, the performance of the classification model was evaluated using 6 metrics, including ACC, Precision, Recall, F1 score, AUROC, and MCC. Subsequently, we predicted the specific value of the RPA using a regression prediction. To this end, we utilized the *R*^2^ and RMSE to assess the performance of the regression model. The calculation formulas for the evaluation metrics are as follows:

$$\mathrm{ACC}=\frac{\mathrm{TP}+\mathrm{TN}}{\mathrm{TP}+\mathrm{TN}+\mathrm{FP}+\mathrm{FN}}$$ (14)

$$\text{Precision}=\frac{\mathrm{TP}}{\mathrm{TP}+\mathrm{FP}}$$ (15)

$$\text{Recall}=\frac{\mathrm{TP}}{\mathrm{TP}+\mathrm{FN}}$$ (16)

$$F1-\text{Score}=2\times \frac{\text{Precision}\times \text{Recall}}{\text{Precision}+\text{Recall}}$$ (17)

$$\mathrm{MCC}=\frac{\mathrm{TP}\times \mathrm{TN}-\mathrm{FN}\times \mathrm{FP}}{\sqrt{\left(\mathrm{TP}+\mathrm{FP}\right)\times \left(\mathrm{TP}+\mathrm{FN}\right)\times \left(\mathrm{TN}+\mathrm{FP}\right)\times \left(\mathrm{TN}+\mathrm{FN}\right)}}$$ (18)

$$R^{2}=1-\frac{\sum_{i=1}^{n}{\left(y_{i}-\hat{y_{i}}\right)}^{2}}{\sum_{i=1}^{n}{\left(y_{i}-\bar{y}\right)}^{2}}$$ (19)

$$\text{RMSE}=\sqrt{\frac{1}{n}\sum_{i=1}^{n}{\left(y_{i}-\hat{y_{i}}\right)}^{2}}$$ (20)

where TP is true positive, TN is true negative, FP is false positive, and FN is false negative.

## Data Availability

Training and test sets for 178 individual proteins in classification and regression prediction tasks, as well as the associated code, are available at the following links: https://github.com/fxh1001/ML_PC.
