# Supplementary material for: Machine Learning Enables Comprehensive Prediction of the Relative Protein Abundance of Multiple Proteins on the Protein Corona
Source: Research (Wash D C). 2024 Sep 25;7:0487. doi: 10.34133/research.0487 (PMC11423712; doi:10.34133/research.0487)
Supplement: Supplementary 1 — Figs. S1 to S6 Tables S1 to S6 [file research.0487.f1.zip › Supplementary Materials.pdf]

Supplementary Materials

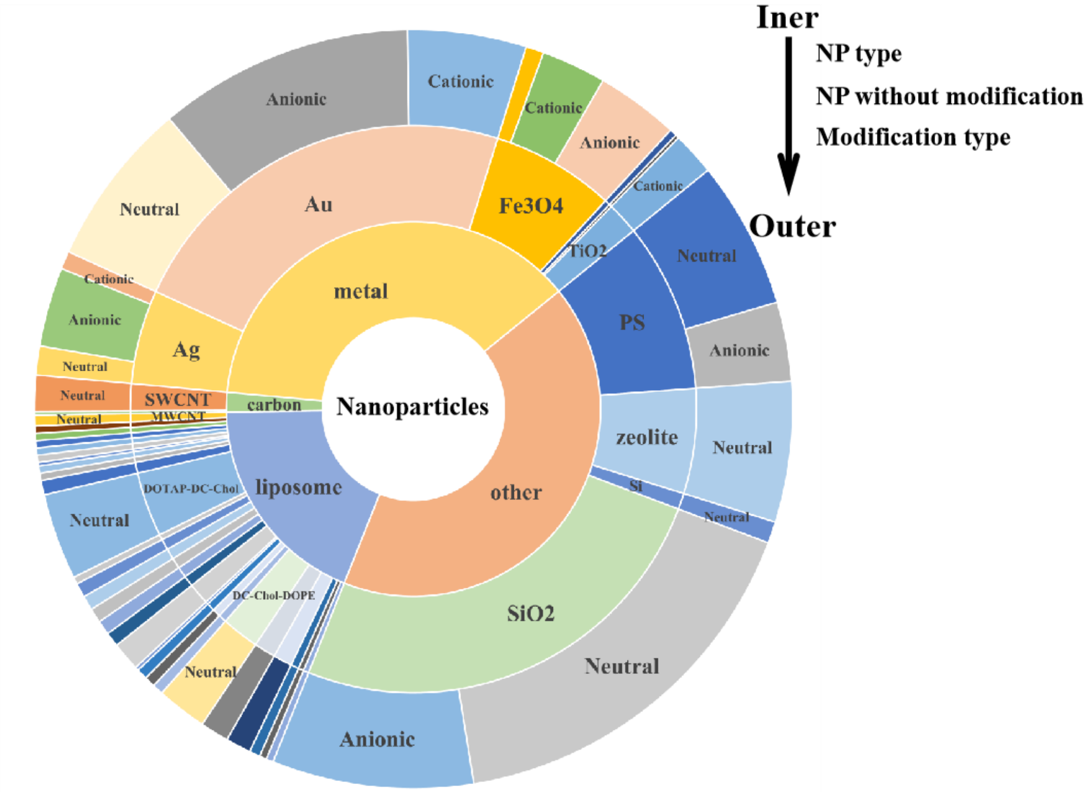

Fig. S1 Abbreviated distribution of nanoparticle attributes: np type, np without modification, modification type in the dataset used.

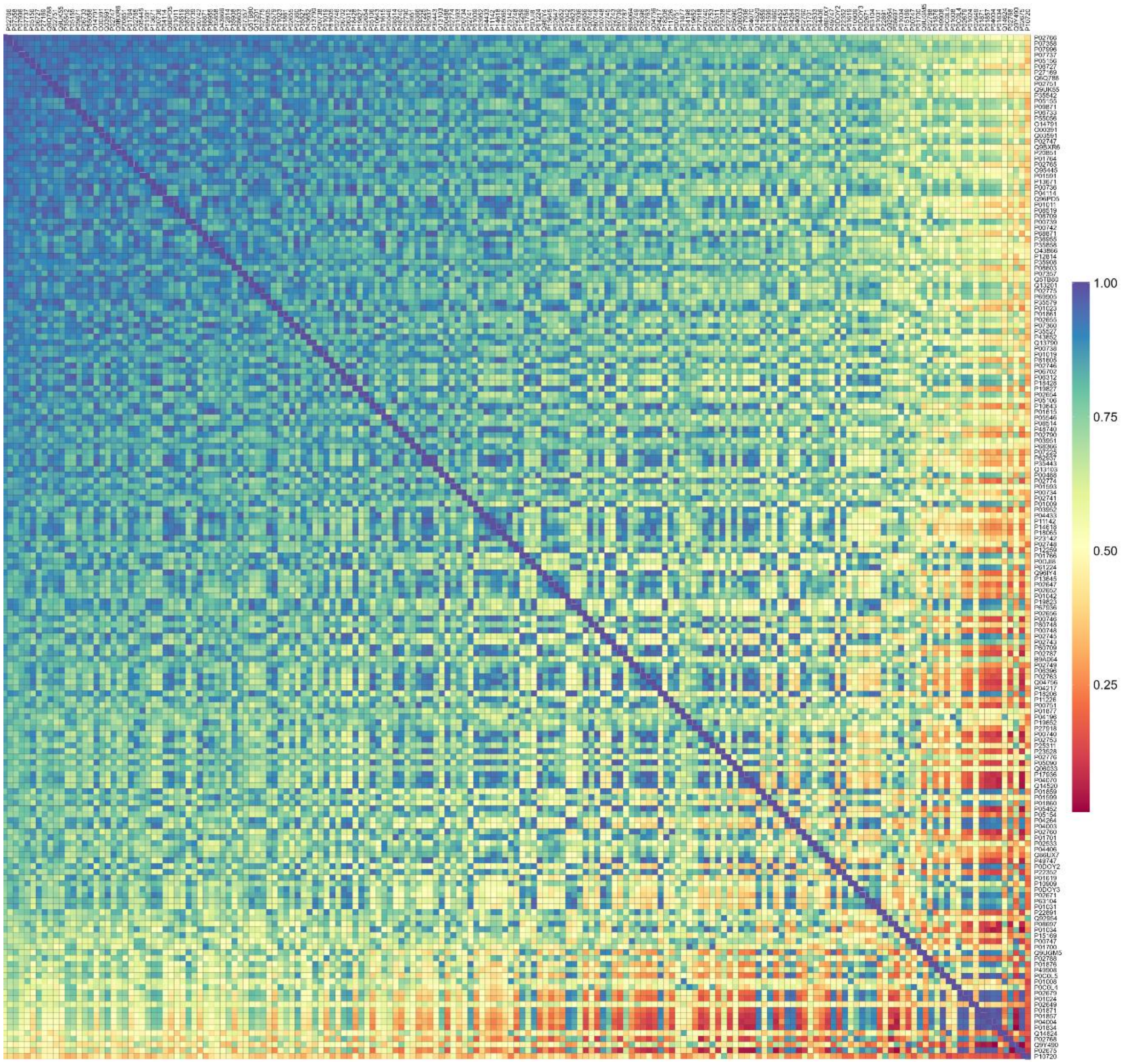

Fig. S2 Heatmap of the correlation between the feature importance of 178 independent proteins corresponding to the ERT baseline models in the classification task (from left to right, top to bottom, arranged in the order of magnitude of the mean value).

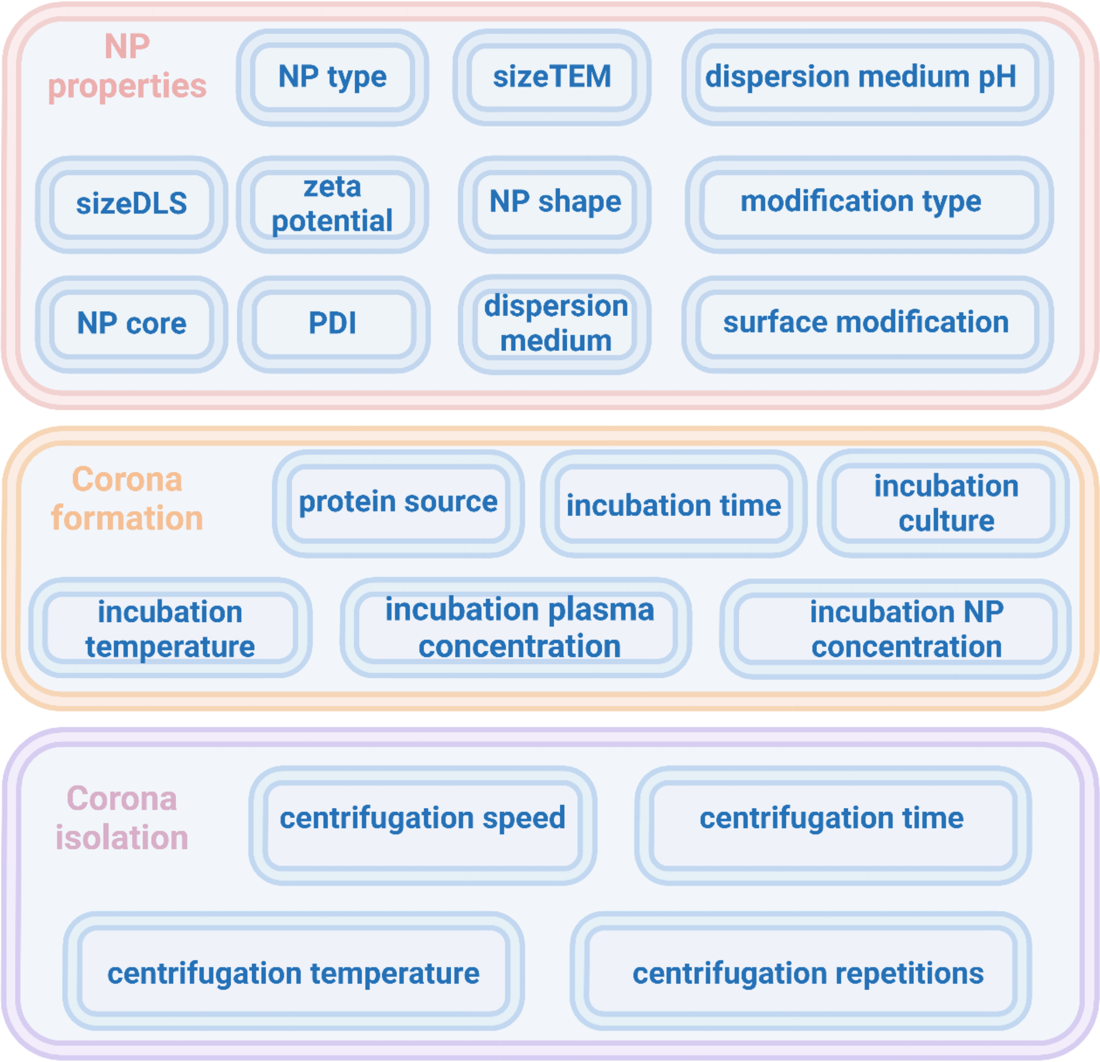

Fig. S3 Detailed names of features in the experimental data used.

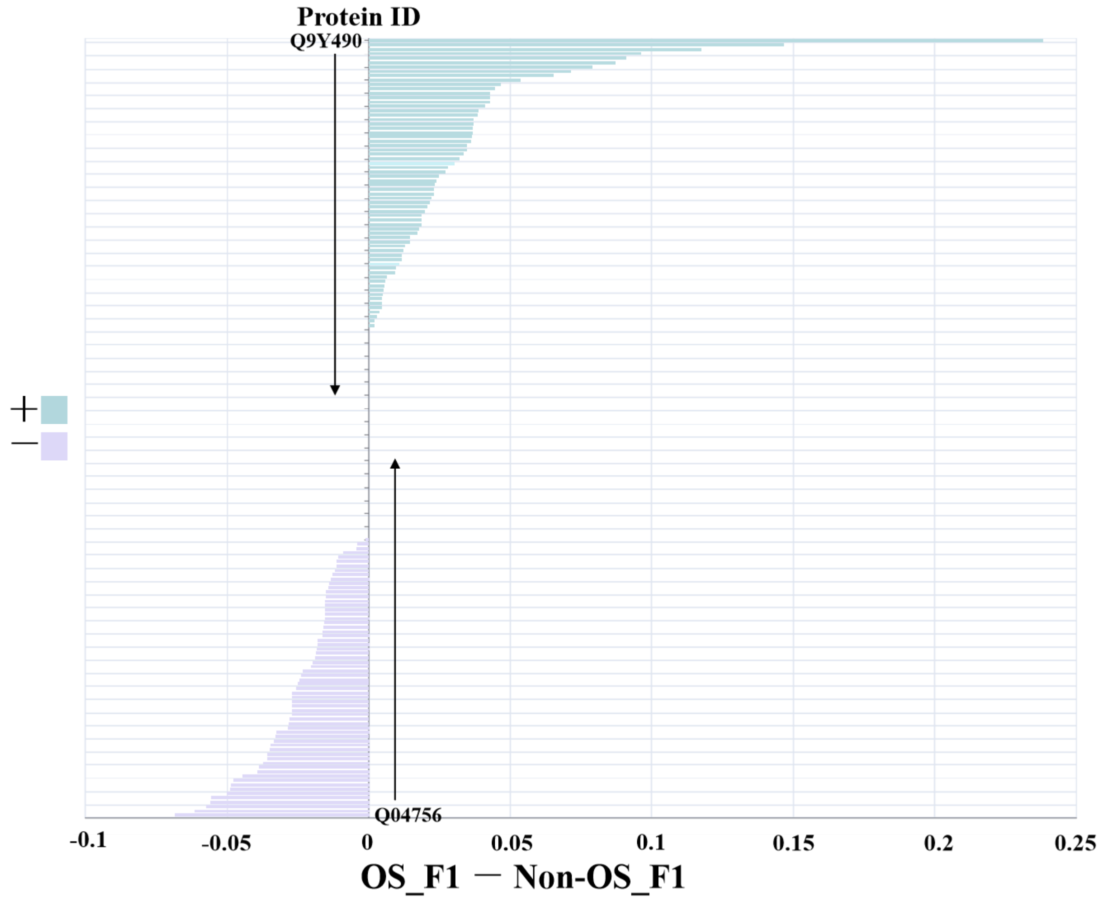

Fig. S4 Gain after oversampling treatment of all 178 individual protein models. The middle section represents multiple protein datasets with an oversampling gain of 0.

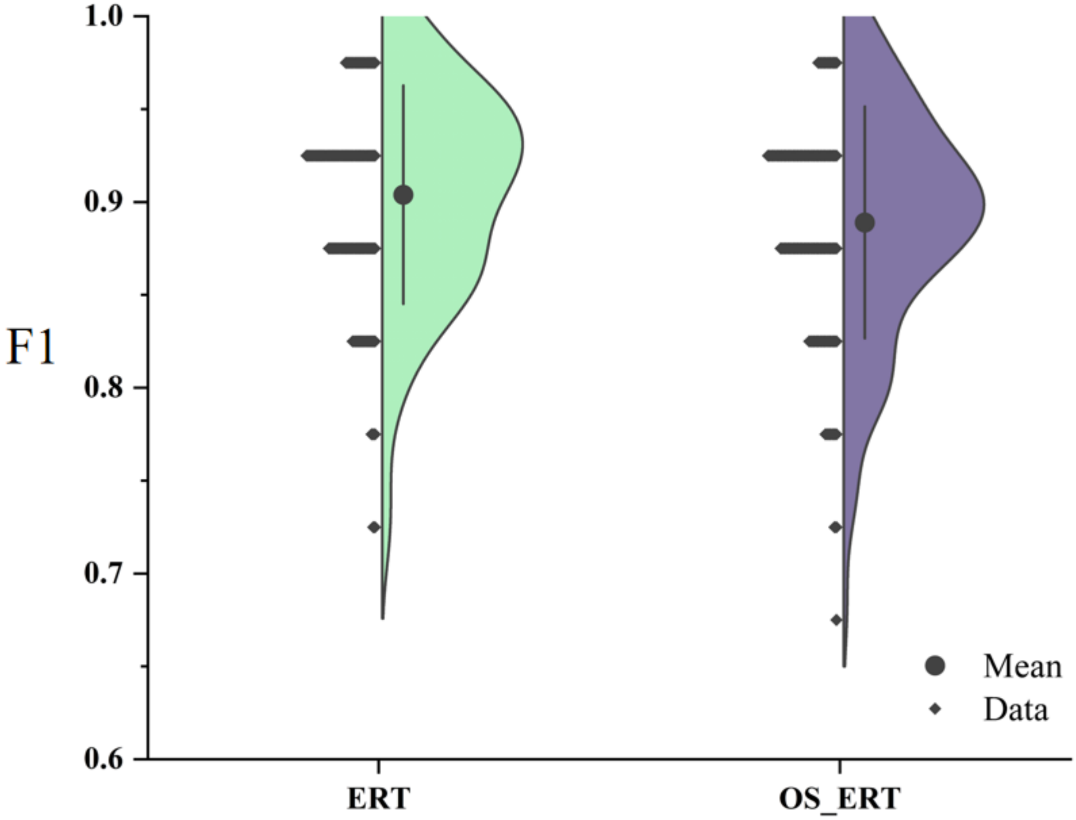

Fig. S5 Comparison of F1 metrics using oversampling and not using oversampling for individual protein models with no gain from oversampling.

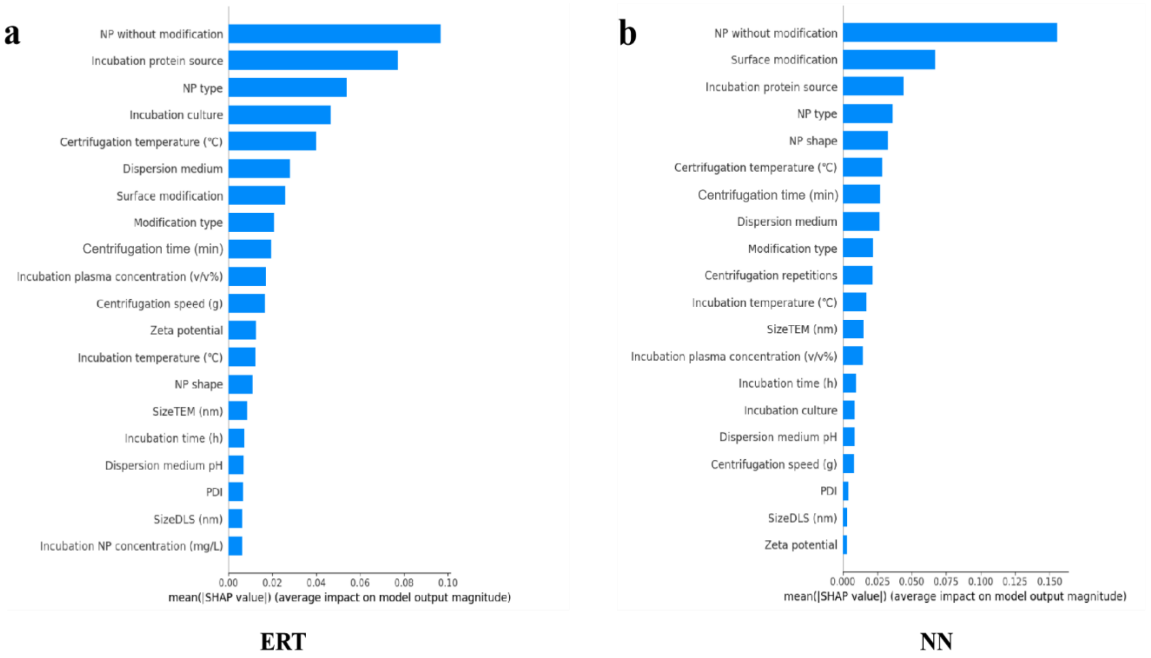

Fig. S6 Preliminary assessment of the importance of the respective features of ERT and NN. a. ERT. **b.** NN.

Tabel S1 Hyperparameter selection for ERTs and its search range.

Hyperparameters	Range
n_estimators	range(50, 2000, step = 25)
min_sample_split	range(2, 13, step = 1)
max_feature	[1, "log2", "sqrt", 16, 32, 64]
max_depth	range(1, 25, step = 2)
bootstrap	[True,False]

Table S2 Average performance of ERT after oversampling and undersampling versus before on 178 independent proteins. Bold values indicate the best results.

	AUROC↑	Recall↑	Precision↑	F1↑	MCC↑	ACC↑
**ERT**	0.969	0.877	**0.915**	0.893	0.811	**0.919**
**after-undersampling**	0.961	0.890	0.850	0.864	0.770	0.894
**after-oversampling**	**0.970**	**0.895**	0.902	**0.896**	**0.818**	**0.919**

Table S3 Comparison of the average performance of methods using undersampling and sample weight changes with and without these methods on these individual proteins using oversampled negative gain. Bold values indicate the best results.

	AUROC↑	Recall↑	Precision↑	F1↑	MCC↑	ACC↑
**Undersampling**	0.962	0.888	0.854	0.866	0.773	0.894
**Sample weight change**	0.966	**0.899**	0.888	0.891	0.802	0.912
**ERT(original)**	**0.969**	0.891	**0.921**	**0.903**	**0.828**	**0.925**

Table S4 Performance comparison of different neural network architectures on protein corona data, including 2-5 layer neural networks (NN) and LSTM. Bold values indicate the best results.

	AUROC↑	Recall↑	Precision↑	F1↑	MCC↑	ACC↑
**2-layer NN (we use)**	**0.921**	**0.857**	0.876	**0.862**	**0.765**	**0.900**
**3-layer NN**	0.906	0.847	0.880	0.860	0.760	0.898
**4-layer NN**	0.891	0.842	0.882	0.857	0.754	0.896
**5-layer NN**	0.883	0.838	**0.885**	0.856	0.750	0.897
**LSTM[48]**	0.904	0.843	0.836	0.834	0.719	0.885

Table S5 Average performance of six machine learning algorithms on validation sets of 178 independent proteins. Bold values indicate the best results.

	AUROC↑	Recall↑	Precision↑	F1↑	MCC↑	ACC↑
**ERT**	**0.960**	0.863	**0.902**	0.878	0.788	0.910
**RF**	0.959	0.867	0.900	**0.879**	**0.790**	**0.911**
**GBDT**	0.952	0.866	0.899	0.878	0.788	0.910
**XGB**	0.956	0.850	0.901	0.870	0.779	0.906
**LGBM**	0.951	0.847	0.895	0.866	0.767	0.902
**NN**	0.956	**0.868**	0.861	0.859	0.760	0.895

Table S6 Average performance of ERT, RF, and GBDT on validation sets of 178 independent proteins in regression prediction. Bolding indicates the best results.

	RMSE ↓	R^2 ↑
**ERT**	**0.675**	**0.358**
**RF**	0.693	0.301
**GBDT**	0.690	0.292
