# Supplementary figures and images for: Machine Learning Enables Comprehensive Prediction of the Relative Protein Abundance of Multiple Proteins on the Protein Corona
Source: Research (Wash D C). 2024 Sep 25;7:0487. doi: 10.34133/research.0487 (PMC11423712; doi:10.34133/research.0487)

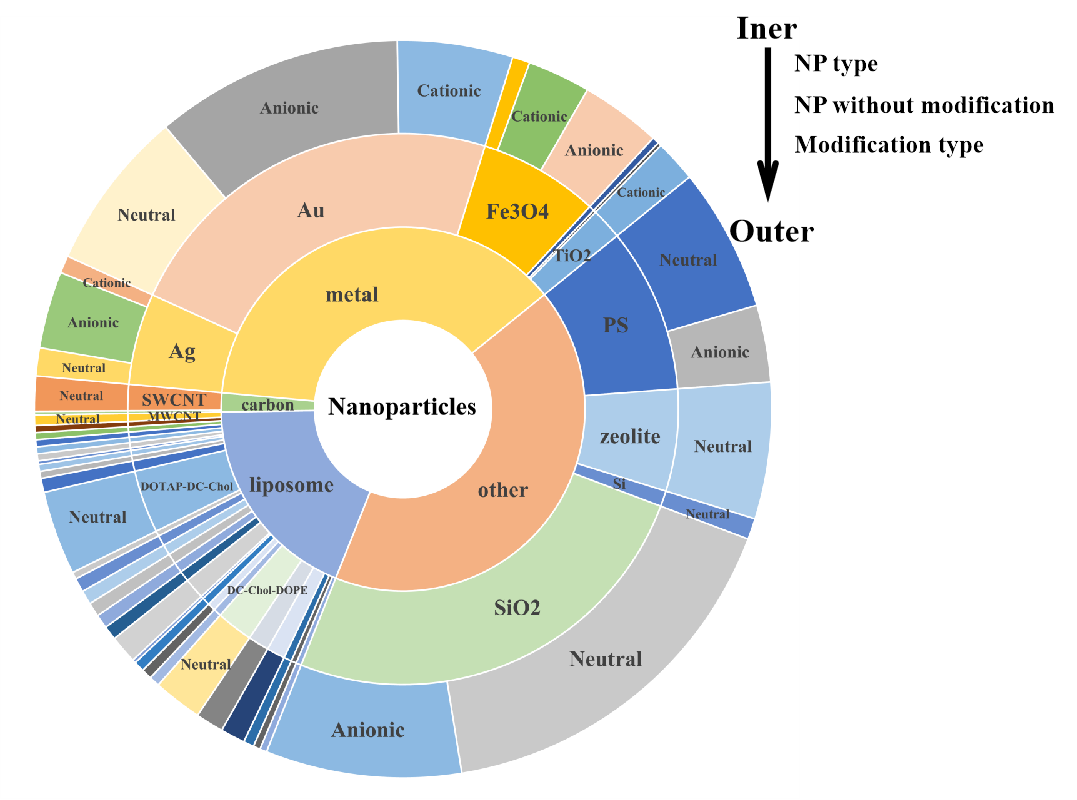

Supplement: Supplementary 1 — Figs. S1 to S6 Tables S1 to S6 [file research.0487.f1.zip › FigureS1.png]

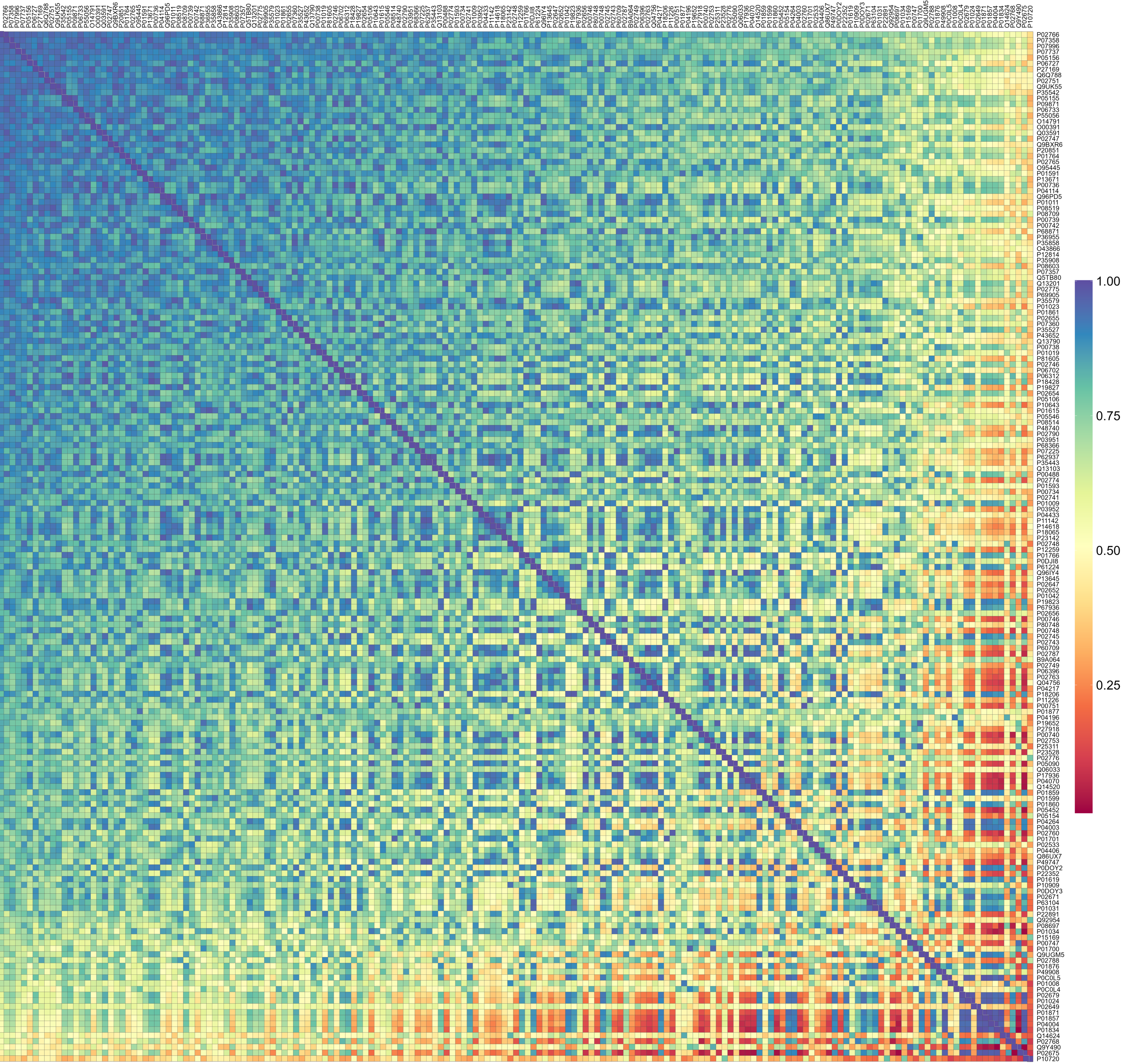

Supplement: Supplementary 1 — Figs. S1 to S6 Tables S1 to S6 [file research.0487.f1.zip › FigureS2.png]

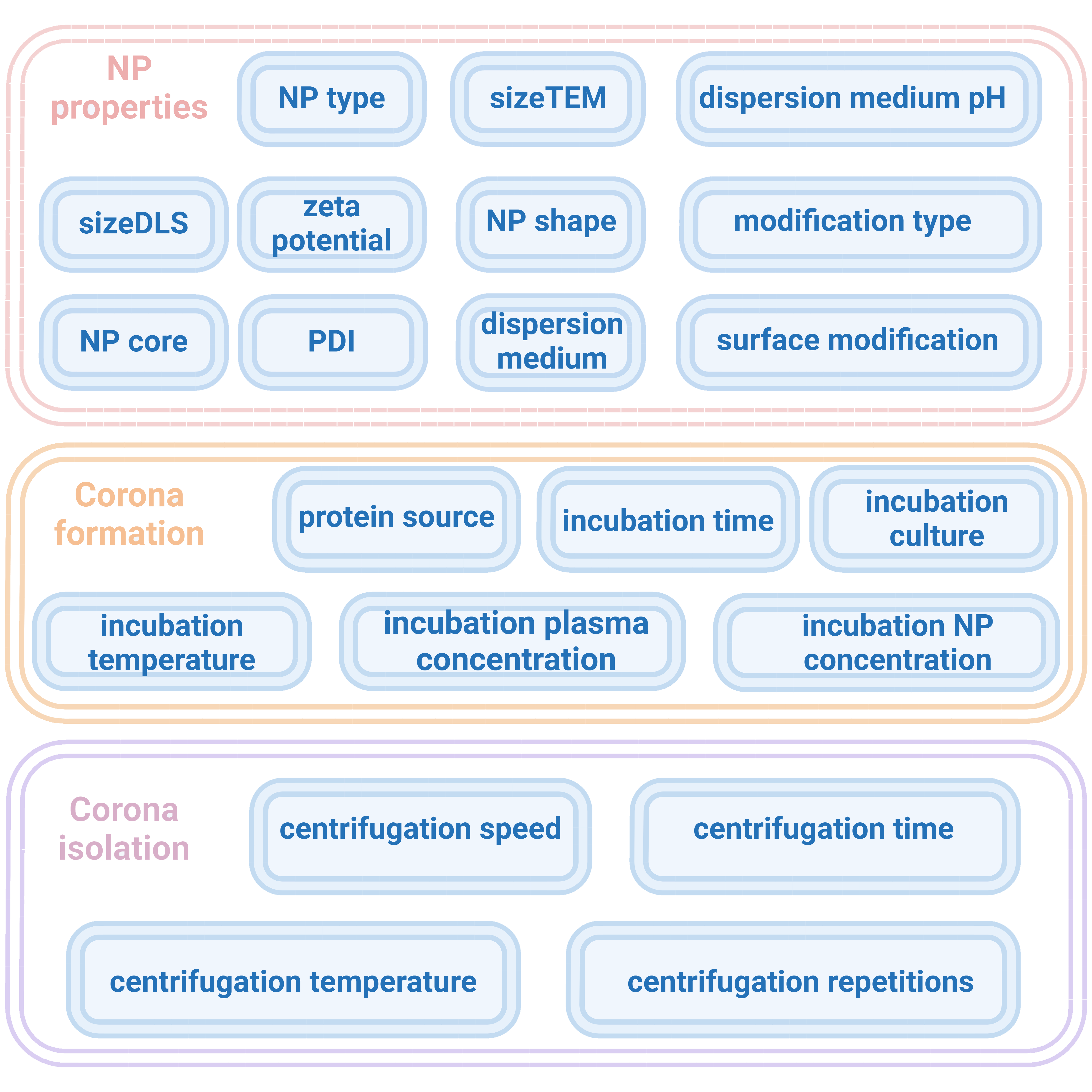

Supplement: Supplementary 1 — Figs. S1 to S6 Tables S1 to S6 [file research.0487.f1.zip › FigureS3.png]

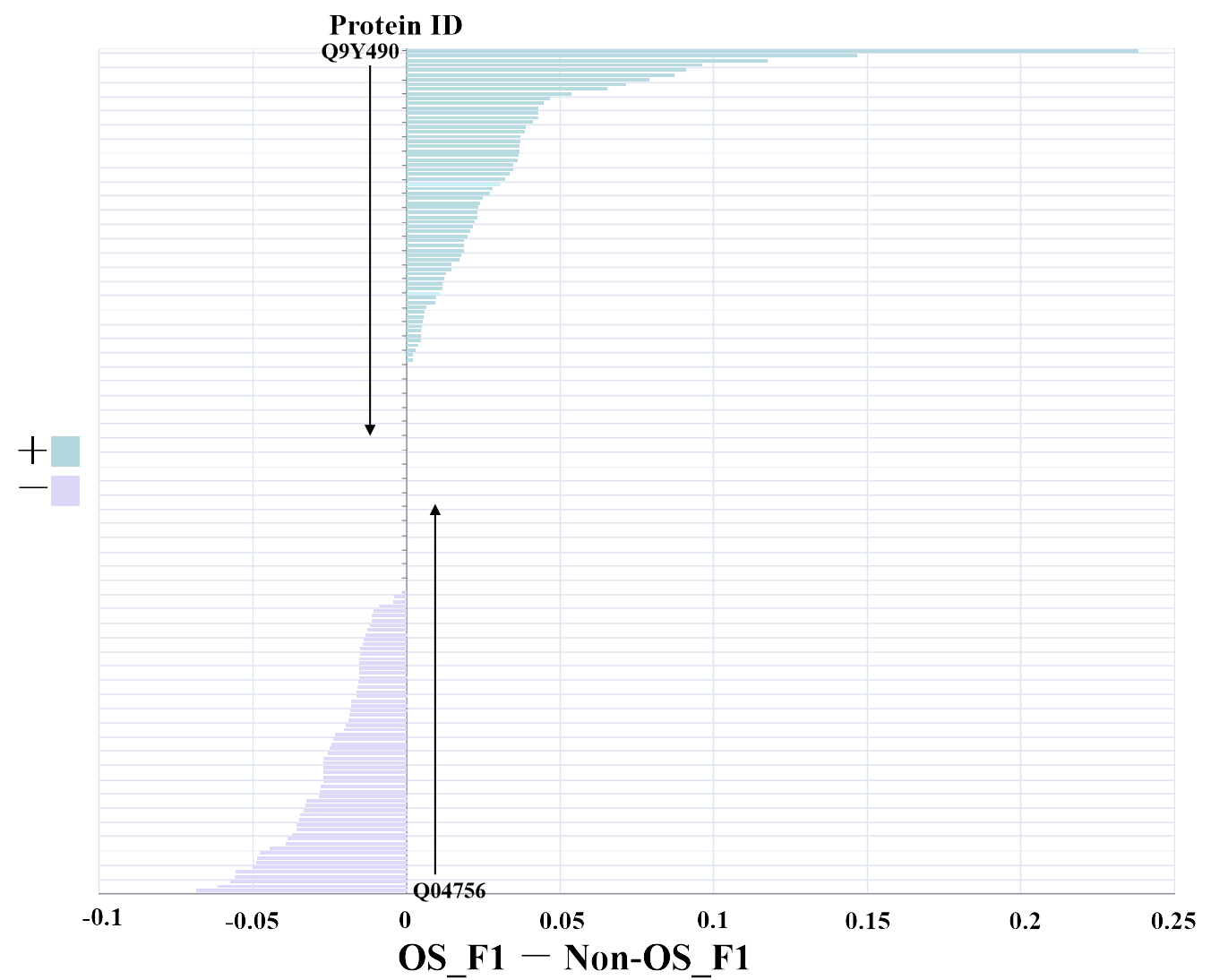

Supplement: Supplementary 1 — Figs. S1 to S6 Tables S1 to S6 [file research.0487.f1.zip › FigureS4.png]

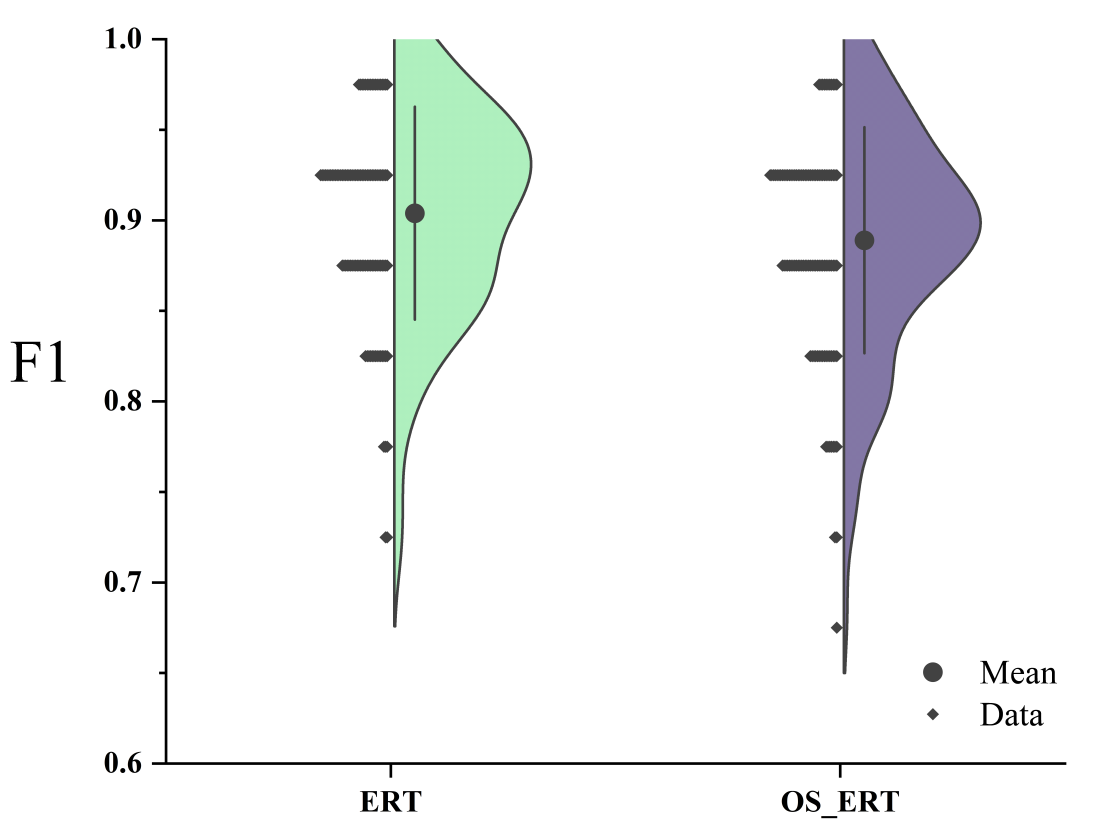

Supplement: Supplementary 1 — Figs. S1 to S6 Tables S1 to S6 [file research.0487.f1.zip › FigureS5.png]

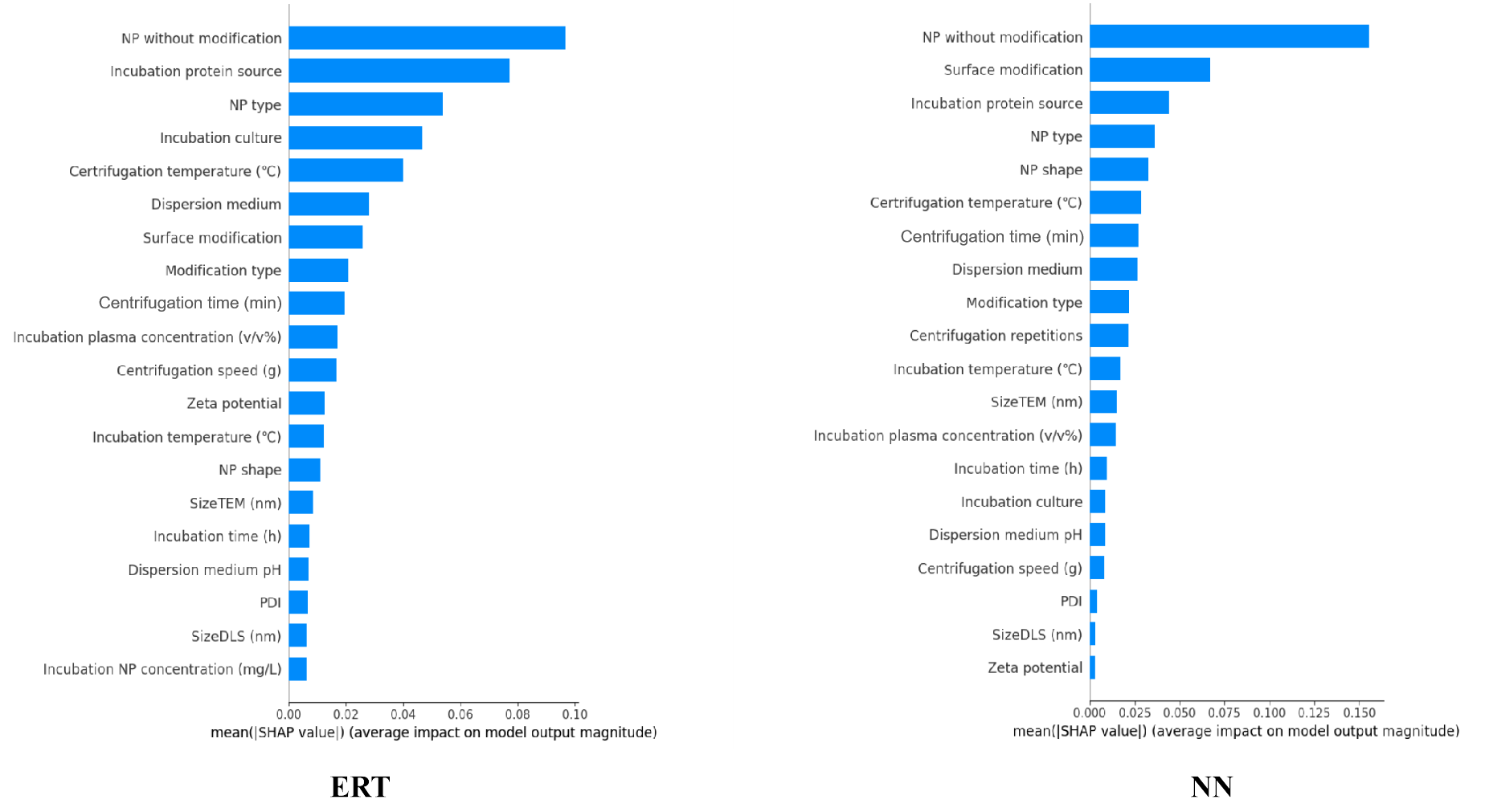

Supplement: Supplementary 1 — Figs. S1 to S6 Tables S1 to S6 [file research.0487.f1.zip › FigureS6.png]
